# Cuticular hydrocarbons for identifying Sarcophagidae (Diptera)

**DOI:** 10.1038/s41598-021-87221-y

**Published:** 2021-04-08

**Authors:** Hannah E. Moore, Martin J. R. Hall, Falko P. Drijfhout, Robert B. Cody, Daniel Whitmore

**Affiliations:** 1 Cranfield Forensic Institute, Cranfield University, Defence Academy of the United Kingdom, Shrivenham, Wiltshire, SN6 8LA UK; 2 Department of Life Sciences, Natural History Museum, Cromwell Road, London, SW7 5BD UK; 3 Chemical Ecology Group, School of Chemical and Physical Science, Keele University, Keele, ST5 5BG England, UK; 4 JEOL USA, Inc. 11 Dearborn Rd., Peabody, MA 01969 USA; 5 Staatliches Museum für Naturkunde Stuttgart, Rosenstein 1, 70191 Stuttgart, Germany

**Keywords:** Biochemistry, Chemical biology

## Abstract

The composition and quantity of insect cuticular hydrocarbons (CHCs) can be species-specific as well as sexually dimorphic within species. CHC analysis has been previously used for identification and ageing purposes for several insect orders including true flies (Diptera). Here, we analysed the CHC chemical profiles of adult males and females of eleven species of flesh flies belonging to the genus *Sarcophaga* Meigen (Sarcophagidae), namely *Sarcophaga africa* (Wiedemann), *S. agnata* Rondani, *S. argyrostoma* Robineau-Desvoidy, *S. carnaria* (Linnaeus), *S. crassipalpis* Macquart, *S. melanura* Meigen, *S. pumila* Meigen, *S. teretirostris* Pandellé, *S. subvicina* Rohdendorf, *S. vagans* Meigen and *S. variegata* (Scopoli). Cuticular hydrocarbons extracted from pinned specimens from the collections of the Natural History Museum, London using a customised extraction technique were analysed using Gas Chromatography–Mass Spectrometry. Time of preservation prior to extraction ranged between a few weeks to over one hundred years. CHC profiles (1) allowed reliable identification of a large majority of specimens, (2) differed between males and females of the same species, (3) reliably associated males and females of the same species, provided sufficient replicates (up to 10) of each sex were analysed, and (4) identified specimens preserved for up to over one hundred years prior to extraction.

## Introduction

The cuticle of insects is covered by an epicuticular lipid wax layer, which is predominantly composed of hydrocarbons (cuticular hydrocarbons or CHCs). The lipid wax layer also consists of fatty acids, alcohols, glycerides, phospholipids and glycolipids^1^. Insect CHCs are composed of long linear chains of hydrogen and carbon atoms, with a chain length typically varying from C17 to C35^2,3^. These compounds are observed in both the saturated and unsaturated forms and can have one or more methyl groups attached at various points along the backbone of their length. In the saturated form, the chain is formed by single bonds, but the CHCs may be linear or branched. In the unsaturated form, one or more double bonds may be present along the length of the chain^3,4^.

Although the primary function of insect CHCs is water-proofing and protection against desiccation and microorganisms^2,5–7^, they also play an important role in intra- and interspecific communication as sexual pheromones, epideictic pheromones and semiochemicals^8^. Besides being used for species and gender recognition in non-social insects^8^, CHCs are used in a number of additional ways in social insects, including nest-mate recognition, caste recognition, thermoregulation, territory marking and as task allocation cues^9,10^. Chemical mimicry, in which inquilines of social insect colonies have evolved CHC profiles identical to those of their hosts, has been found in several termitophilous and myrmecophilous species^11,12^. The role of cuticular hydrocarbons in sexual behaviour and mate choice has been confirmed for representatives of several insect orders^13–15^ and has been particularly well studied in some Diptera species, in which CHCs seem to facilitate courtship behaviour (see Wicker-Thomas^16^). Additional functions of CHCs as epideictic pheromones (e.g., regulating larval mosquito population densities or involved in host marking by parasitoids) or kairomones have been documented in the literature^17^.

The remarkable variability of CHCs and their role in courtship and mate choice as well as species recognition indicate that they are likely at the basis of speciation processes, including in sympatry^18–20^. Studies on non-social insects in which CHCs are central to species recognition and sexual selection have suggested a saltational model for the evolution of divergent chemical profiles, strictly correlated to speciation events^14,20^, whereas a more gradual evolution model has been suggested for ant CHCs, particularly when qualitative traits are analysed^7,20^. Regardless of their mechanisms of evolution, species-specific differences in CHC profiles represent a valid identification tool for species across several insect groups, also for closely related ones^14,21–24^, and provide chemotaxonomic characters for classification^25^. The reader is referred to reviews on the general role and function of CHCs in insects by Howard and Blomquist^8^ and Drijfhout et al.^26^.

The study of CHCs as contact pheromones in Diptera and their potential use for species identification were summarised by Wicker-Thomas^16^. Use of CHCs in Diptera identification has been largely focused on groups of medical or agricultural importance or on genetic model organisms such as *Drosophila Fallén*^23,27–30^. Chemical identification of specimens has been tested on Diptera of forensic importance such as blow flies (Calliphoridae), where CHC analysis can be the only means of species determination when dealing with, for example, small, degraded fragments of the empty puparia that are no longer identifiable through morphological or molecular analysis. CHC identification of adult blow flies has been successfully carried out by Roux et al.^31^, Pechal et al.^32^, Barbosa et al.^33^ and Butterworth et al.^34^, whereas Moore et al.^35^ were able to identify first instar larvae and puparia of these flies through their CHC profiles. Cuticular chemical analysis can also be used to determine the age of specimens, as adults, larvae, puparia or throughout their ontogeny^35–37^, as well as determine their degree of sexual maturity^38^.

The flesh fly genus *Sarcophaga* Meigen contains over 900 species worldwide, the taxonomic relationships of which are still very incompletely known^39^. The identification of these flies is challenging, which can be problematic if misidentifications occur in applied sectors such as medicine and forensics^40,41^. Existing keys provide only a very partial coverage of the species in this genus, especially for immature stages and females. Therefore, morphological identification ultimately relies on the input of the few taxonomic specialists for the family. Alternative morphology-based identification methods have also been explored for adults^42–44^ and larvae^45^, but with a very limited species coverage. COI barcodes represent an excellent alternative to morphology for the identification of *Sarcophaga* flies^46–48^. However, DNA extraction is not always possible and reference libraries are still incomplete.

Flesh flies (Diptera: Sarcophagidae) have been much less studied than blow flies with regard to their cuticular hydrocarbon profiles, despite also including species of medical and forensic importance. CHCs of the common North American species *Sarcophaga bullata* (Parker) were studied both qualitatively and quantitatively throughout all development stages from larva to adult^49^. Cvačka et al.^50^ included this species in a study testing the matrix-assisted laser desorption/ionization mass spectrometry method for detection of CHCs. Ye et al.^51^ compared the cuticular hydrocarbons extracted from puparial exuviae of selected necrophagous flies, including two flesh fly species. More recently, Braga et al.^52^ used CHCs for the identification of four South American sarcophagid species of forensic importance based on extracts from puparia.

The primary objective of this study was to test the efficacy of CHCs as an identification tool for adult Sarcophagidae, using a customised extraction protocol adapted to pinned museum specimens. We also wanted to assess whether the CHC profiles of the studied species were sexually dimorphic and whether males and females of the same species could be associated through their hydrocarbon profiles. Our dataset also allowed us to assess the stability of CHCs over varying preservation times since the death of the specimens.

## Results

### *Sarcophaga* CHC profiles

#### Males

The males of the eleven *Sarcophaga* species yielded chemical profiles of 61 peaks with percentage peak areas exceeding 0.5% of the total. The hydrocarbons consisted of *n*-alkanes (15%), alkenes (25%), methyl branched hydrocarbons (52%) and unknown CHCs (8%), with chain lengths ranging from C22:H to C35:H (Table S1). The double bond positions were not determined for alkenes and alkadienes within this study. In general, the odd-numbered *n*-alkanes had much larger peak areas, with heptacosane (C27:H) dominating the profiles in most species, followed by nonacosane (C29:H). The main exception to this was within the profiles of *S. teretirostris*, where 11 + 13-Methyl C27 was the most dominant, followed by C27:H and 11 + 13-Methyl C25. The males of only four species yielded alkenes within their chemical profiles. *Sarcophaga africa* and *S. pumila* each yielded the same alkene, a diene, within their profiles: C27:2. *Sarcophaga agnata* and *S. melanura* yielded eight and nine alkenes, respectively.

Within the male dataset, analyses of historical (i.e., collected 117 to 5 years before CHC extraction) specimens of three species, *Sarcophaga subvicina*, *S. variegata* and *S. carnaria*, were repeated with more recent samples from specimens collected in 2018. Since CHCs very slowly degrade with time, the concentration of historical profiles was significantly lower than that of recent samples. However, the differences observed were primarily quantitative rather than qualitative, as seen in Tables S1 and S2. Analysed specimens of *Sarcophaga crassipalpis* were collected only in 2018.

#### Females

The chemical profiles of the female *Sarcophaga* specimens yielded 58 peaks with percentage peak areas exceeding 0.5% of the total. The hydrocarbons consisted of *n*-alkanes (16%), alkenes (16%), methyl branched hydrocarbons (59%), unknown CHCs (7%) and aldehydes (3%), with chain lengths ranging from C21:H to C31:H (Table S2). The odd-numbered *n*-alkanes generally had larger peak areas, with heptacosane dominating the profiles in most species except *S. vagans* and *S. variegata*, where 11 + 13-Methyl C25 and 11 + 13-Methyl C27 were the dominant peaks, respectively. The chemical profile of *S. pumila* yielded trimethyl C24 as the most abundant peak, closely followed by 3,x-DiMethyl C25. As with the males, female *S. melanura* also yielded more alkenes (eight in total) than any other species. The only alkene not shared with the female that was visible within the male profile of this species was an additional C29:2. The *Sarcophaga agnata* females had a chemical profile that was made up of four alkenes and three other species (*S. africa*, *S. teretirostris*, and *S. pumila*) yielded a low number of alkenes (1 or 2).

### Chemical identification of *Sarcophaga* spp.

#### Males

Males of all eleven species were analysed with the objective of identification using support vector machine (SVM) classification, which showed 98.3% Leave-One-Out Cross Validation (LOOCV) accuracy for all males (historical and recent), with only two misclassifications. One historical *S. carnaria* sample was misclassified as *S. variegata* and one *S. melanura* sample was misclassified as *S. africa*.

The compounds listed in Table S1 were selected as features for chemometric analysis. Analysis of Variance (ANOVA) was carried out for these features for each of the subset of species and for the full dataset. Only those compounds with ANOVA p ≤ 0.05 for each classification were used (Table S3).

#### Females

Females of ten species were analysed using the LOOCV method. A LOOCV accuracy of 97.1% was observed, with two misclassifications out of 69 samples by SVM analysis of the data for all females. One *S. carnaria* was misclassified as *S. argyrostoma* and one *S. variegata* sample was misclassified as *S. teretirostris*, respectively. Table S3 displays the compounds used for LOOCV analysis.

#### Males and females combined

**Figure 1:**
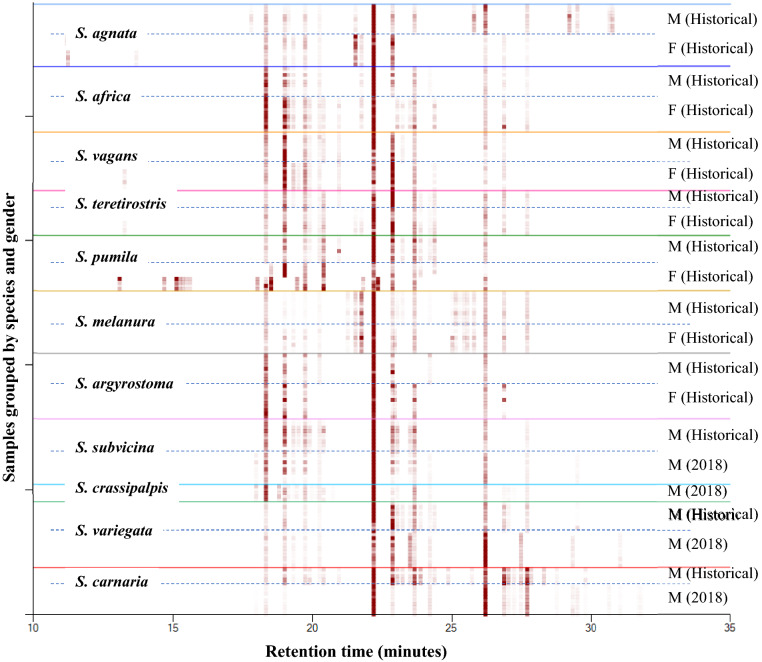
Heat map of all 60 + compounds from the males and females of all *Sarcophaga* species, showing gender, time and species-dependent differences in the chromatograms. The historical samples cover a time period which ranges over 117 years. The x-axis represents the retention time and the chromatographs are grouped along the y-axis by species. The heat map is a visual aid, enabling multiple chromatographs to be efficiently stacked for comparison in a small vertical space, in which darker spots represent larger peak areas. For example, the most abundant compound, with a retention time of around 22.1 min on the heat map, is C27 (Table S1, peak number 23, Table S2, peak number 35). Some individual *S. pumila* female samples exhibited chromatograms that appeared different on the heat map. Nevertheless, SVM correctly classified all of these specimens as *S. pumila*. The compounds used for classifying are presented in Table S3.

Due to a smaller sample set of morphologically reliably identified females of *S. carnaria* (n = 3), *S. subvicina* (n = 3) and *S. variegata* (n = 2), these were not included in the dataset for males and females combined. This dataset therefore included males of 11 species (recent and historical samples) and females of seven species. These results are displayed in the heat map in Fig. 1. Differences between the chromatographic data are evident when viewed as a heat map, where the x-axis represents the retention time and the chromatographs are grouped along the y-axis by species. The heat map enables multiple chromatographs to be efficiently stacked for comparison in a small vertical space, in which darker spots represent larger peak areas.

Support vector machine classification with Leave-One-Out Cross Validation gave a classification accuracy of 98.3% for both species and gender, with only 3 misclassifications out of 177 samples. Specifically, one *S. melanura* female was misclassified as a *S. agnata* male, one *S. carnaria* male as a *S. variegata* male and one *S. melanura* male as a *S. africa* male. As an additional validation test, 30% of the samples were selected at random (while including at least one member of each species) and removed from the training set of all males and females. The removed samples were treated as unknowns. There were three errors: one sample out of the 50 “unknowns” was classified as the incorrect species and gender (a *S. vagans* female was classified as a *S. teretirostris* male) and two *S. variegata* females were misclassified as *S. variegata* males, giving a validation accuracy of 96% (Table S6).

### Comparison of recent and historical samples

**Figure 2:**
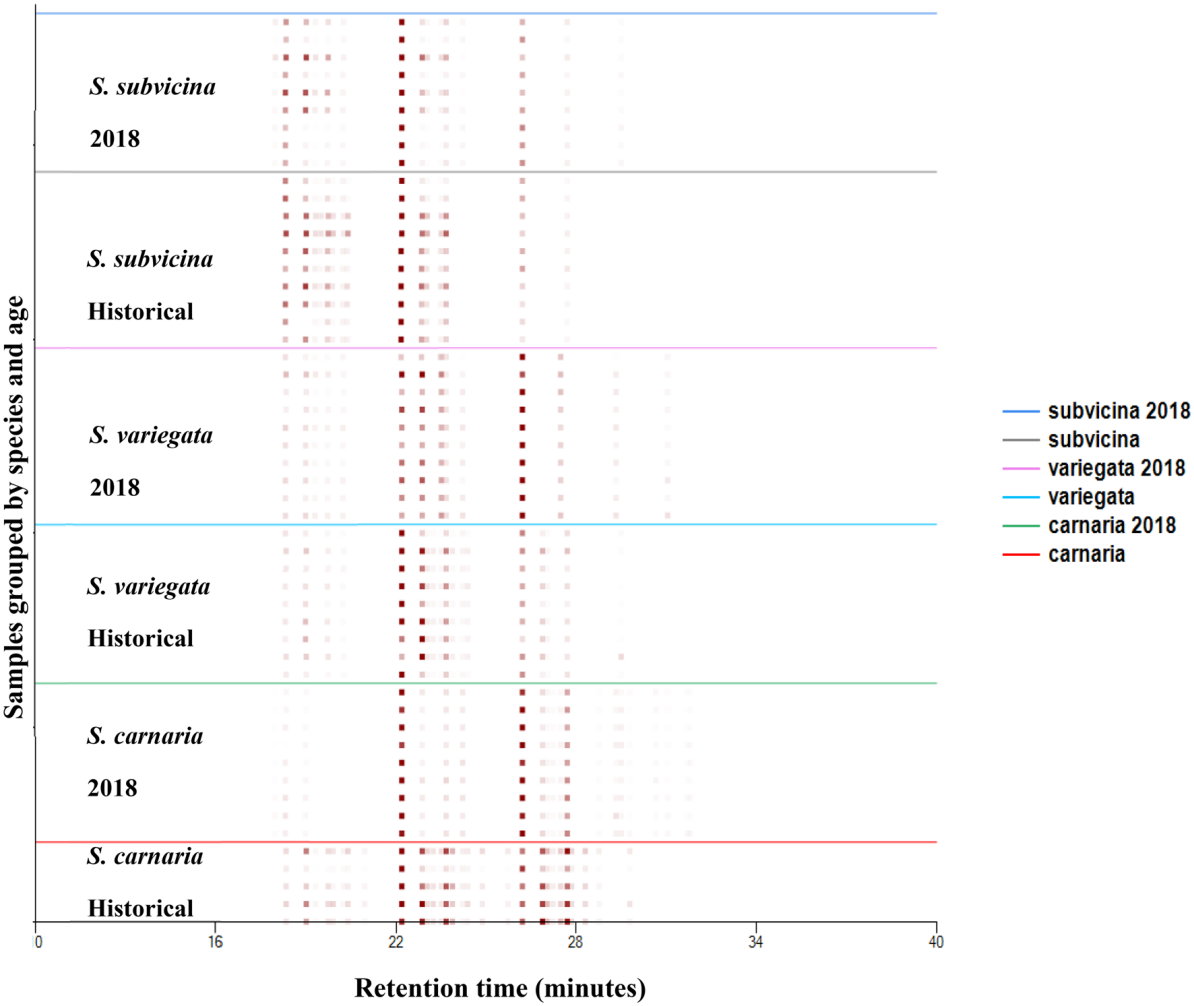
A heat map of differences between cuticular hydrocarbon profiles of historical and recent (2018) specimens of three species of *Sarcophaga* (males only). The intensity of the red spots are proportional to the abundance of each compound with the observed retention time.

**Figure 3:**
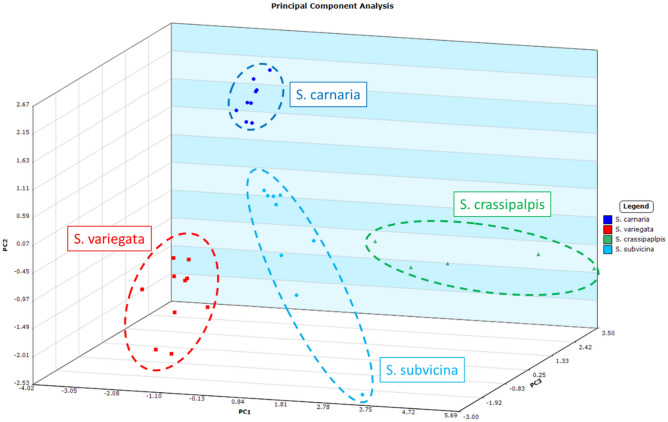
Principal component analysis for samples from the males of four species (*Sarcophaga* *subvicina*, *S. variegata*, *S. crassipalpis* and *S. carnaria*) collected and sampled in 2018. The first three principal components cover 43.4%, 23.5% and 14.3% of the variance, respectively, for a total of 81.2% variance. Factor loadings for the first three principal components are listed in the Supporting Data (Table S4).

Males of four species (*S. carnaria*, *S. crassipalpis*, *S. subvicina* and *S. variegata*) were collected from the wild in 2018 as examples of recently collected specimens. These four species can clearly be distinguished by their hydrocarbon profiles as is evident from the heat map in Fig. 2 and the Principal Component Analysis (PCA) graph in Fig. 3. LOOCV for SVM classification gives 100% accuracy for assignment of each sample. The compounds used for PCA are given in Table S3.

**Figure 4:**
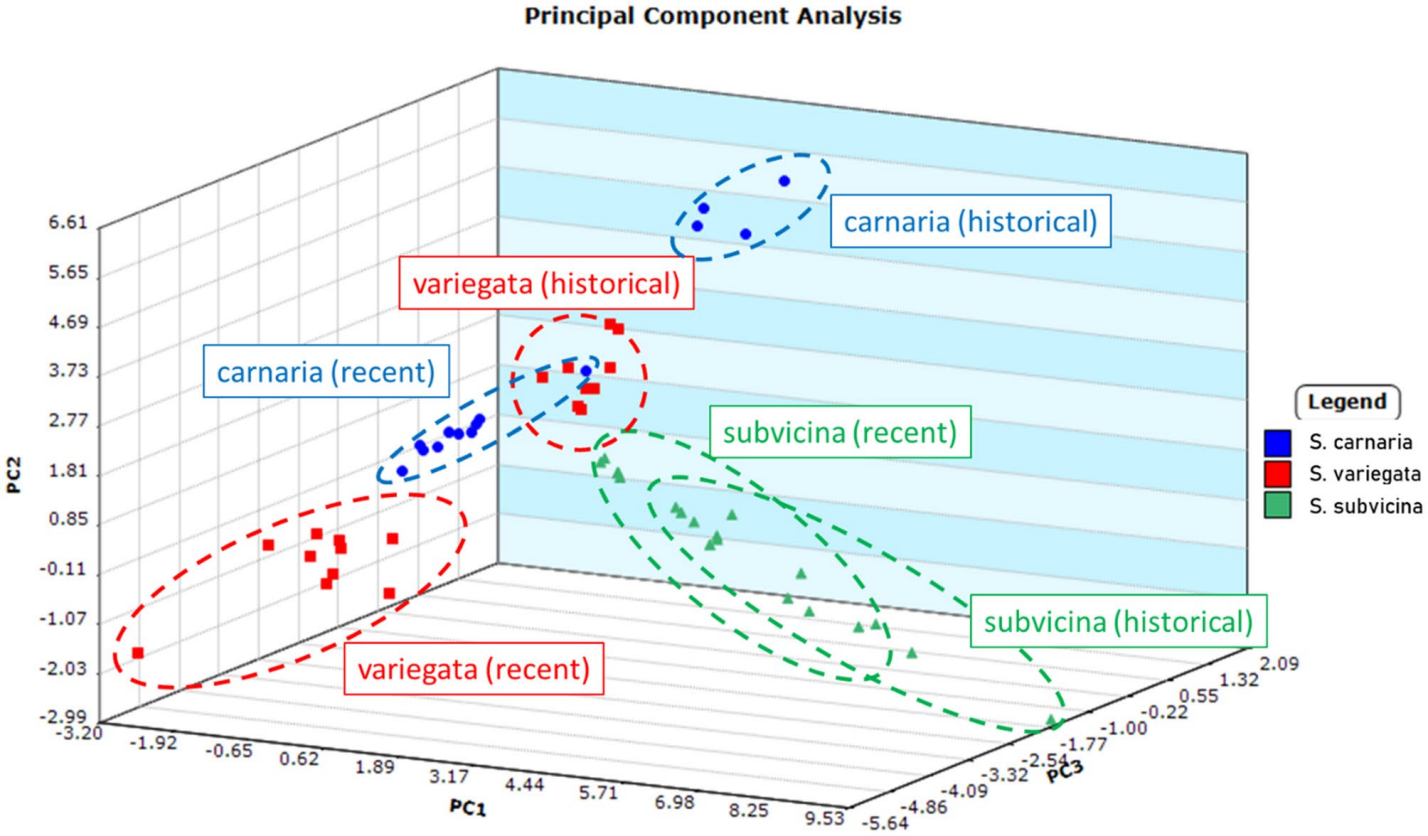
Principal component analysis for male samples from three species (*Sarcophaga subvicina*, *S. variegata* and *S. carnaria*) collected recently (2018), compared to samples from males of the same three species collected historically (5 + years prior to extraction). The compounds used for PCA are given in Table S3. The first three principal components cover 39.3%, 23.3% and 14.4% of the variance, respectively, for a total of 77.0% variance. Factor loadings for the first three principal components are listed in the Supporting Data (Table S5).

A comparison was made of recent (2018) and historical (5 + years) specimens using three species (*S. carnaria*, *S. subvicina* and *S. variegata*). The three-dimensional PCA graph (Fig. 4) created using the compounds in Table S3 shows some differences between samples of these three species obtained from specimens collected in 2018 compared to more historical samples for the same species. Intraspecific differences were less than interspecific differences, regardless of age. Despite these differences, LOOCV using SVM classification only resulted in one misclassification: one of the historical *S. subvicina* samples was assigned as a recent *S. variegata*, giving a classification accuracy of 98%. We also analysed the data by creating separate classes for each of the three species according to whether they were recent or historical. LOOCV with SVM classification correctly assigned all but one sample to the correct species and correct historical age, the exception being one of the recent *S. carnaria* samples, misclassified as a historical *S. variegata*.

**Figure 5:**
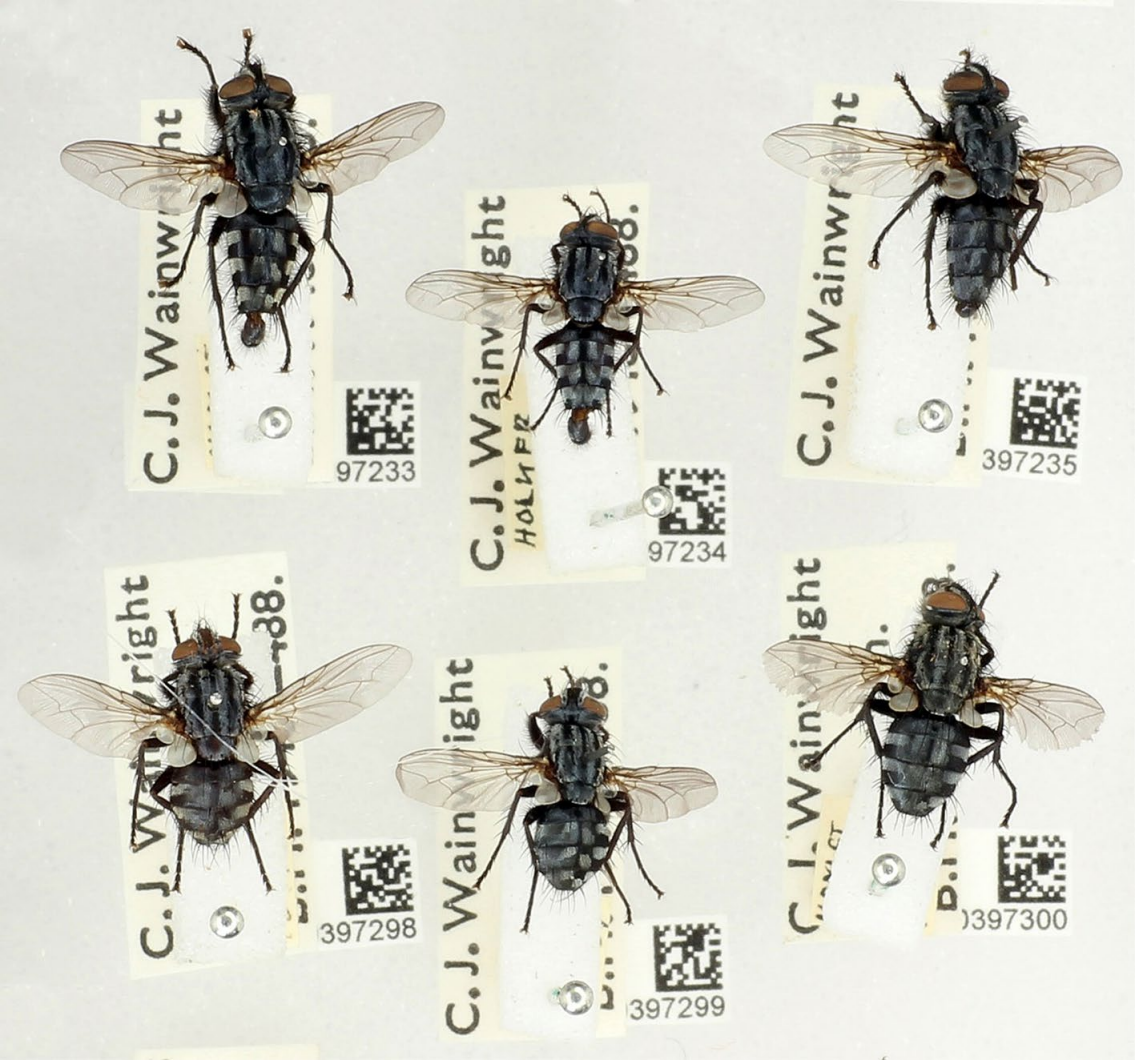
Image showing three *Sarcophaga agnata* males (top row) and three *S. agnata* females (bottom row), 6 months after the specimens were extracted with hexane to examine their cuticular hydrocarbon profiles.

After the specimens were extracted with hexane, their appearance did not alter and they could still be identified using the usual morphological criteria (Fig. 5).

## Discussion

Traditional morphological identification of adult *Sarcophaga* flies relies on the careful preparation and detailed observation of species-specific characters of the genitalia, whereas the morphological identification of larval stages requires specialist preparation of specimens for analysis using a combination of different techniques^53,54^. Furthermore, some species are very similar and can easily be confused. This can lead to incomplete or incorrect identifications, which in turn lead to incorrect diagnoses and errors in applied fields such as forensics and medicine^38,55^. Females of closely related species are often impossible to identify morphologically and, in many of the less common species, are unknown or have not been associated with the males.

DNA barcoding using the standard fragment of the COI mitochondrial gene has proved successful for species’ delimitation, identification and discovery in Sarcophagidae^48,56^, although some species are problematic, possibly due to introgression^46^. However, there can also be limitations to molecular techniques due to DNA decomposition, which makes it challenging to amplify extracts from older, incorrectly preserved specimens^51,57^.

Numerous publications have highlighted the benefits of chemotaxonomy for the identification of various insect species^28,51,58,59^. Several recent studies have used cuticular hydrocarbons for chemical identification of species of Calliphoridae (blow flies), a family closely related to flesh flies. Roux et al.^31,60^ analysed the CHCs of three species throughout ontogeny from the egg to the adult stage and found that interspecific differences were mainly quantitative rather than qualitative. Moore et al.^61^ successfully used CHCs to identify first instar larvae of the three forensically important species *Lucilia sericata* (Meigen), *Calliphora vicina* Robineau-Desvoidy and *C. vomitoria* (Linnaeus), whereas Barbosa et al.^33^ chemically identified three Neotropical species based on adult extracts. Recently, Butterworth et al.^34^ analysed intra- and interspecific variation in CHC profiles of ten species of *Chrysomya* Robineau-Desvoidy.

Our study was the first to test CHC chemical identification across a large sample of adult Sarcophagidae. Previously, Ye et al.^51^ included two species of *Sarcophaga* in their CHC study of puparial exuviae of flies of forensic importance, whereas Braga et al.^57^ examined the cuticular hydrocarbon profiles of four species of Sarcophagidae of forensic importance in South America—*Peckia chrysostoma* (Wiedemann), *P. intermutans* (Walker), *P. lambens* (Wiedemann) and *Sarcophaga ruficornis* (Fabricius)—using empty puparial cases, which can be the only Diptera remains left at older crime scenes and which can be difficult to identify morphologically. Braga et al.^57^ analysed their specimens using GC–MS, with the aim of using the chemical profiles to distinguish between the four species. The specimens yielded chain lengths ranging from C23 to C33 composed of alkanes, methyl and di-methyl alkanes and two alkenes. The insect specimens were reared in the laboratory in a controlled environment and, by applying Bray–Curtis distances to the data sets, Braga et al. could successfully discriminate between the four species analysed^57^. Our results show that chemical analysis of cuticular hydrocarbons shows great promise (98% accuracy) for the identification of both males and females of the selected species of *Sarcophaga*, provided that a sufficient number of replicate samples is analysed. This result is especially relevant for females, which can be even more challenging to identify than males using morphological criteria. Males and females of the same species could also be associated via their chemical profiles with sufficient replicates (n > 3), which could be used as a tool for gender association in species for which the females are unknown or in species described based on female type specimens. Chemotaxonomy as a tool for the identification of species is still in its infancy and therefore has its limitations. There are several factors that can lead to changes in a CHC profile (such as age, gender, sexual maturity), which need to be considered. Despite this, with careful planning and enough replicates, these variations are often seen in the statistical analysis. The reader is encouraged to consult the excellent review on advantages and limitations in using cuticular hydrocarbon profiles as a taxonomic tool by Kather and Martin^62^.

We expanded on the work of Ye et al.^51^ and Braga et al.^57^ by using dry adult museum specimens collected up to 117 years before analysis. CHC analysis is a non-destructive technique, enabling the specimens to be returned to the collections in an undamaged state^63,64^. Cuticular hydrocarbons are notoriously unreactive and the long hydrocarbon chain makes these compounds non-volatile at environmental temperatures, hence their degradation is expected to be slow, allowing for stable CHC profiles and making this technique a potential complimentary identification tool, sometimes even the only tool in cryptic species complexes where DNA has degraded. The stability of cuticular hydrocarbon profiles provides the potential for specimen identification to be established chemically from museum collections many years after collection. Carlson first established this when he examined the chemical profiles extracted from museum specimens of cockroaches, honey bees and tsetse flies^64^, demonstrating a non-destructive means of identification from historical specimens. The results from the eleven Sarcophagidae species presented and analysed in this paper show great potential for chemical identification of individuals belonging to this family of Diptera, including that of very old specimens collected over 100 years ago. Although the samples collected and extracted in 2018 yielded a much higher concentration of CHCs (a similar phenomenon was found for specimens of four hornet species which were between 3 and 13 years old^63^), CHCs of the historical samples were still stable and detectable and could still be statistically distinguished from the chemical profiles of other species.

Chemometric analysis with the *Mass Mountaineer* software has been widely applied for plant chemotaxonomy^65–75^ and animal chemotaxonomy^76,77^. Musah and coworkers used these chemometric methods for speciation of necrophagous insect eggs based upon amino acid profiles^78^, and also for species-level identification of fly larvae feeding on decomposing remains^79^. Musah et al.^66^ identified Calliphoridae species from hydrocarbons extracted from puparial casings.

Although males and females of the *Sarcophaga* species analysed herein could be associated through their CHC profiles, they also showed marked sexual dimorphism. Sexually dimorphic cuticular hydrocarbons have been documented in several insect groups, although their function may not always be related to sexual behaviour. Ortiz-Dominguez et al.^80^ linked sexually dimorphic CHCs to a role in sexual recognition in scarab beetles; Thomas and Simmons^15^ reviewed sexual dimorphism in cuticular hydrocarbons, concluding that it is mainly driven by sexual selection. Butterworth et al.^81^ showed that sexually dimorphic CHCs did not play a role in sexual attraction in blow flies of the species *Chrysomya varipes* (Macquart), but the same authors recently suggested that changes in the CHC composition in adults of that species may play a role in signalling the sexual maturity of individuals^34,38^. It is possible that sexually dimorphic CHCs play a similar role in *Sarcophaga* species, as well as a role in sexual recognition. Males of these flies behave territorially, performing short, darting flights to intercept passing individuals. This behaviour was documented in detail in male hill-topping aggregations by Povolný and Vachá^82^, who described males darting after other males (usually resulting in brief aerial fights sometimes including attempted copulation), females (resulting in attempted or successful copulation) or even small stones thrown above their resting perch. It is not clear whether recognition of a conspecific female, leading to successful copulation, is the result of chemical stimuli or of other types of stimuli. Thomas^83^ observed attempted copulation with females of different species or even males before a conspecific female was found, suggesting that chemical stimuli may not be prevalent, and that female receptivity might also be important for successful copulation—see also Blackith and Blackith^84^. Clearly, further behavioural research is needed to assess the role of sexually dimorphic CHCs in sarcophagids.

This proof-of-concept research project provides a platform for expansion of identifying flesh fly species based on their hydrocarbon profiles, also using museum collections when needed. Our future studies aim to determine if the chemical profiles can be applied to ageing of the different life stages of forensically relevant Sarcophagidae species such as *S. argyrostoma* and *S. crassipalpis*, as has been demonstrated with Calliphoridae^32,60,85,86^.

## Methods

### Insect materials

In this study, we extracted CHCs from dry-preserved adults of eleven species *Sarcophaga* collected between 1901 and 2018, using a customised extraction protocol. Samples were analysed using Gas Chromatography–Mass Spectrometry (GC–MS) and chemometric analysis.

We analysed a total of 185 specimens belonging to eleven species of *Sarcophaga* collected from various localities in the United Kingdom over a time span varying from 117 years to 8 weeks before chemical extraction (Tables S7 and S8). The majority of specimens are deposited in the Natural History Museum (NHM) collection in South Kensington (London), whereas a subsample of specimens from Harmondsworth was kindly provided by M. Harrow (London). All NHM specimens were dry-pinned for long-term preservation in standard entomological drawers. All specimens were identified in 2017 and 2018 using a preliminary version of the key to British species by Whitmore, Dupont and Falk (https://osf.io/vf5r6/).

### Sample preparation

For each species, ten male and ten female flies were extracted where possible, with the exception of *S. carnaria*, *S. subvicina* and *S. variegata*, for which only three female specimens identified to species level (through association with males with which they had been collected as mating pairs) were extracted. Each specimen was hand-held by its pin over a glass vial; 350 μL of hexane were dripped over the specimen from a syringe and the resulting extract was collected in a glass vial as it came off the fly. The vials were left uncapped until the hexane had fully evaporated. They were then washed out with 200 μL of hexane, which were transferred to a 300 μL spring bottom glass insert placed within 2 mL GC vials. These were allowed to dry down completely and stored dry in a refrigerator at 4 °C until they were required for analysis. The dried extract was reconstituted in 30 μL of hexane before GC–MS analysis using an autosampler.

### Chemical analysis: Gas Chromatography–Mass Spectrometry

Chemical analysis of all extracts was carried out on an Agilent Technologies 6890 N Network GC with a split/splitless injector at 250 °C, a Restek Rxi-1MS capillary column (30 m × 0.25 mm ID, 0.25 μm film thickness) and coupled to an Agilent 5973 Network Mass Selective Detector. The GC was coupled to a computer and the data were processed with the Agilent Chemstation software. Elution was carried out with helium at 1 mL/min. The oven temperature was programmed to be held at 50 °C for 2 min then ramped to 200 °C at 25 °C/min, then from 200 to 260 °C at 3 °C/min and finally from 260 to 320 °C at 20 °C/min, where it was held for 2 min. The mass spectrometer was operated in Electron Ionisation mode at 70 eV, scanning from 40 to 500 amu at 1.5 scans s^−1^. Hydrocarbons were identified using a library search (NIST08), the diagnostic fragmented ions and the Kovats indices.

### Statistical analysis

For each species, up to ten male and ten female adult flies were used in the statistical analyses, due to some specimens not yielding a detectable chemical profile (Table S9). From the GC chromatographs of eleven species, the peak areas of 35 compounds, including *n*-alkanes, alkenes and methyl branched alkane compounds, were used for statistical analyses. By removing those compounds with low variance, feature selection is an important component of model generation for classification methods. For each compound, the means were calculated for all classes. Analysis of variance (ANOVA) was calculated for the two classes that showed the greatest difference in means. If the compound did not show a statistically significant difference between those classes, it was eliminated.

Statistical analysis was applied once to all males separately, once to all females separately and once to males and females combined. A utility program was created to extract text files for each sample analysis from the summary spreadsheet. The text files, consisting of data pairs for retention time and peak area, one pair per line, were used by the *Mass Mountaineer* software, which uses supervised and unsupervised learning algorithms to identify patterns in data (*massmountaineer.com*), to create the heat map, carry out ANOVA analysis for feature selection, and perform chemometric analysis and Leave-One-Out Cross Validation (LOOCV) for each classification method.

Principal component analysis (an unsupervised learning method) was used to visualize species-specific differences in the data. After evaluating several classification models, Support Vector Machine (SVM) classification was determined to be an effective supervised learning method. Thus, validation was carried out with SVM for each model (see^87^ for further reading on SVM). Classification accuracy for LOOCV was reported using SVM classification. That is, a sample was removed from the training set and treated as an unknown. The classifier was recalculated from the remaining examples in the training set and used to assign a class to the “unknown”. This was repeated for each sample until all samples had been classified. The calculated classifications were compared to their known, correct, class membership to determine the LOOCV accuracy. The total concentration of hydrocarbons could vary widely with sample age and storage. For the heat map all peak areas in each chromatogram were normalized to the largest peak area, which was assigned a value of 100%. For PCA and SVM, peak areas were further normalized to the sum of peak areas for the compounds selected as features.

## Supplementary Information

Supplementary Information
