# Supplementary material for: Cuticular hydrocarbons for identifying Sarcophagidae (Diptera)
Source: Sci Rep. 2021 Apr 8;11:7732. doi: 10.1038/s41598-021-87221-y (PMC8032779; doi:10.1038/s41598-021-87221-y)
Supplement: Supplementary file 1 — Supplementary Information [file 41598_2021_87221_MOESM1_ESM.docx]

**Supplementary Data**

**Cuticular hydrocarbons for identifying Sarcophagidae (Diptera)**

Hannah E. Moore^a*^, Martin J.R. Hall^b^, Falko P. Drijfhout^c^, Robert B. Cody^d^, Daniel Whitmore^e^

^a^ Cranfield Forensic Institute, Cranfield University, Defence Academy of the United Kingdom, Shrivenham, Wiltshire, SN6 8LA, UK, * [h.e.moore@cranfield.ac.uk](mailto:h.e.moore@cranfield.ac.uk)

^b^ Department of Life Sciences, Natural History Museum, Cromwell Road, London, SW7 5BD, UK

^c^ Chemical Ecology Group, School of Chemical and Physical Science, Keele University, ST5 5BG, England, UK

^d^JEOL USA, Inc. 11 Dearborn Rd., Peabody MA 01969 USA

^e^Staatliches Museum für Naturkunde Stuttgart, Rosenstein 1, 70191 Stuttgart, Germany

Table S1: List of the compounds extracted from historical and recent (2018) male *Sarcophaga* flies with the total percentage of each compound present, ± the percentage standard deviation for each species (ND = Not Detected). The recent samples are presented in shaded columns.

|  |  |  | ***S. africa*** | ***S. agnata*** | ***S. argyrostoma*** | ***S. carnaria*** | ***S. melanura*** | ***S. pumila*** | ***S. subvicina*** | ***S. teretirostris*** | ***S. vagans*** | ***S. variegata*** | ***S. carnaria*** | ***S. subvicina*** | ***S. variegata*** | ***S. crassipalpis*** |
| --- | --- | --- | --- | --- | --- | --- | --- | --- | --- | --- | --- | --- | --- | --- | --- | --- |
|  |  |  | ***n=9*** | ***n=9*** | ***n=9*** | ***n=5*** | ***n=10*** | ***n=8*** | ***n=10*** | ***n=5*** | ***n=9*** | ***n=9*** | ***n=5*** | ***n=9*** | ***n=9*** | ***n=5*** |
| **RT** | **Pk #** | **Pk ID** | % | % | % | % | % | % | % | % | % | % | % | % | % | % |
| 17.981 | 1 | C23 | ND | ND | ND | ND | ND | ND | ND | ND | ND | ND | 0.37±0.77 | 1.14±0.93 | ND | 2.76±7.42 |
| 18.799 | 2 | 13+15-Methyl C23 | ND | ND | ND | ND | ND | ND | ND | ND | ND | ND | ND | ND | ND | 4.23±8.42 |
| 19.354 | 3 | 9+11-Methyl C23 | ND | ND | ND | ND | ND | ND | ND | ND | ND | ND | ND | ND | ND | 0.44±0.58 |
| 19.45 | 4 | 3-Methyl C23 | ND | ND | ND | ND | ND | ND | ND | ND | ND | ND | ND | ND | ND | 1.01±2.19 |
| 19.969 | 5 | C24 | ND | ND | ND | ND | ND | ND | ND | ND | ND | ND | ND | ND | ND | 0.63±0.81 |
| 20.139 | 6 | 11+13-Methyl C24 | ND | ND | ND | ND | ND | ND | ND | ND | ND | ND | ND | ND | ND | 0.53±0.67 |
| 20.731 | 7 | 9+11+13-Methyl C24 | ND | ND | ND | ND | ND | ND | ND | ND | ND | ND | ND | ND | ND | 0.44±0.61 |
| 17.806 | 8 | C25:1 | ND | 1.30±1.90 | ND | ND | ND | ND | ND | ND | ND | ND | ND | ND | ND | ND |
| 18.333 | 9 | C25 | 24.96±29.29 | 5.78±3.43 | 22.20±21.49 | 1.89±1.65 | 6.82±8.09 | 24.96±5.27 | 17.83±21.57 | 4.59±4.62 | 8.44±6.29 | 3.07±2.83 | 1.88±3.55 | 14.22±11.91 | 3.98±4.64 | 22.55±9.95 |
| 19.006 | 10 | 11+13-Methyl C25 | 5.91±4.69 | 2.85±4.35 | 7.73±7.18 | 4.25±4.56 | 0.57±0.30 | 5.91±12.67 | 10.24±8.59 | 12.05±12.77 | 22.15±17.50 | 4.40±3.65 | 0.93±1.04 | 5.83±6.13 | 3.03±1.84 | 6.72±16.22 |
| 19.141 | 11 | 9-Methyl C25 | ND | 0.83±0.77 | ND | ND | ND | ND | ND | ND | ND | ND | ND | ND | ND | 1.09±2.29 |
| 19.127 | 12 | 7-Methyl C25 | 1.81±1.22 | ND | ND | ND | ND | 1.81±0.00 | ND | ND | ND | ND | ND | ND | ND | ND |
| 19.316 | 13 | 5-Methyl C25 | 0.84±0.57 | ND | ND | 0.38±0.40 | 0.51±0.57 | 0.84±0.00 | 1.50±1.13 | 1.02±1.00 | ND | ND | ND | 0.87±0.67 | 0.98±0.66 | ND |
| 19.5 | 14 | 11,x-DiMethyl C25 | ND | ND | ND | ND | ND | ND | 0.96±0.73 | ND | ND | ND | ND | ND | ND | ND |
| 19.736 | 15 | 3-Methyl C25 | 2.32±1.81 | 2.16±2.98 | 3.61±2.98 | 1.43±1.39 | 0.68±0.79 | 2.32±4.01 | 4.85±4.47 | 6.29±6.21 | 5.51±3.84 | 1.80±1.19 | ND | 2.61±1.90 | 1.12±0.73 | 5.17±4.13 |
| 19.9 | 16 | 5,x-DiMethyl C25 | ND | ND | ND | 1.24±1.70 | ND | 0.00±1.26 | 1.10±0.64 | 1.20±1.39 | 1.00±0.76 | ND | ND | ND | ND | ND |
| 20.251 | 17 | C26 | 1.57±1.56 | 0.84±0.49 | 1.94±1.79 | 0.47±0.41 | 0.67±0.58 | 1.57±1.61 | 1.78±1.88 | 0.75±0.42 | 1.33±1.03 | 0.79±0.66 | ND | 1.48±1.33 | 1.36±1.63 | 1.97±1.29 |
| 20.398 | 18 | 3,x-DiMethyl C25 | ND | ND | ND | 1.38±1.21 | ND | 0.00±8.23 | 1.42±0.70 | 4.65±5.10 | 2.23±1.75 | ND | ND | ND | ND | 1.46±4.13 |
| 20.948 | 19 | 12,14Methyl C26 | ND | ND | ND | 0.51±0.51 | ND | 0.00±6.28 | ND | 1.00±1.31 | 2.01±2.14 | ND | ND | ND | ND | ND |
| 21.26 | 20 | C27:2 | ND | ND | ND | ND | 1.67±2.40 | ND | ND | ND | ND | ND | ND | ND | ND | ND |
| 21.537 | 21 | C27:1 | ND | 1.61±2.27 | ND | ND | 5.17±6.74 | ND | ND | ND | ND | ND | ND | ND | ND | ND |
| 21.753 | 22 | C27:1 | 0.76±0.61 | ND | ND | ND | 14.52±18.44 | 0.76±0.00 | ND | ND | ND | ND | ND | ND | ND | ND |
| 22.19 | 23 | C27 | 40.51±40.43 | 29.71±18.12 | 43.77±44.14 | 16.90±20.14 | 33.23±31.59 | 40.51±24.54 | 33.53±36.02 | 15.92±12.20 | 23.52±28.94 | 31.50±38.31 | 30.65±32.51 | 43.41±44.72 | 14.92±10.19 | 30.08±14.98 |
| 22.865 | 24 | 11+13-Methyl C27 | 5.47±4.09 | 4.69±5.66 | 6.13±6.18 | 9.26±9.90 | 3.06±2.03 | 5.47±11.62 | 6.75±4.93 | 26.95±25.23 | 19.74±19.58 | 18.37±13.87 | 2.18±1.84 | 3.12±2.29 | 14.86±10.24 | 1.69±3.79 |
| 23.037 | 25 | 7-Methyl C27 | ND | ND | ND | 1.55±1.16 | 0.67±0.34 | ND | 2.20±1.34 | ND | ND | 2.05±1.50 | ND | 0.77±0.48 | ND | 0.96±1.71 |
| 23.235 | 26 | 5-Methyl C27 | ND | ND | ND | 1.47±1.30 | 0.86±0.64 | 0.00±1.28 | ND | ND | 1.27±1.19 | 1.40±0.86 | ND | ND | ND | ND |
| 23.505 | 27 | Unknown HC | ND | ND | ND | 1.11±0.85 | ND | ND | 1.68±1.22 | ND | ND | 1.12±0.83 | ND | 0.82±0.68 | 10.34±9.19 | ND |
| 23.665 | 28 | 3-Methyl C27 | 6.14±5.50 | 3.86±4.59 | ND | 7.61±6.25 | 4.32±3.47 | 6.14±9.80 | 4.83±3.61 | 8.83±7.45 | 5.63±5.36 | 9.08±6.26 | 3.50±3.02 | 3.65±2.40 | 3.58±4.57 | 8.41±9.77 |
| 23.879 | 29 | Unknown HC | ND | ND | ND | 2.89±1.94 | ND | 0.00±2.31 | ND | 1.20±1.39 | ND | 1.18±0.85 | ND | ND | ND | ND |
| 24.215 | 30 | C28 | 0.67±0.63 | ND | 1.48±1.32 | 0.91±1.01 | 0.43±0.33 | 0.67±1.16 | ND | 0.55±0.48 | ND | 1.22±1.20 | 3.05±3.82 | 1.36±1.65 | 1.69±1.67 | 0.81±1.06 |
| 24.383 | 31 | Unknown HC | ND | ND | ND | 1.24±1.27 | ND | 0.00±4.70 | ND | 2.62±3.16 | ND | 1.33±0.99 | ND | ND | ND | ND |
| 29.324 | 32 | 2-Methyl C28 | ND | ND | ND | ND | ND | ND | ND | ND | ND | ND | 1.68±2.73 | ND | 1.73±1.52 | ND |
| 24.87 | 33 | 12,14-Methyl C28 | ND | ND | ND | 1.07±0.86 | ND | ND | ND | ND | ND | ND | ND | ND | ND | ND |
| 25.02 | 34 | C29:2 | ND | ND | ND | ND | 0.70±0.92 | ND | ND | ND | ND | ND | ND | ND | ND | ND |
| 25.141 | 35 | C29:2 | ND | ND | ND | ND | 3.54±3.55 | ND | ND | ND | ND | ND | ND | ND | ND | ND |
| 25.294 | 36 | C29:2 | ND | ND | ND | ND | 0.85±0.75 | ND | ND | ND | ND | ND | ND | ND | ND | ND |
| 25.445 | 37 | C29:1 | ND | ND | ND | ND | 2.12±2.15 | ND | ND | ND | ND | ND | ND | ND | ND | ND |
| 25.558 | 38 | C29:1 | ND | ND | ND | ND | 2.19±1.98 | ND | ND | ND | ND | ND | ND | ND | ND | ND |
| 25.719 | 39 | Unknown HC | ND | ND | ND | 0.71±0.62 | ND | ND | ND | ND | ND | ND | ND | ND | ND | ND |
| 25.796 | 40 | C29:1 | ND | 5.74±7.40 | ND | ND | 2.89±3.32 | ND | ND | ND | ND | ND | ND | ND | ND | ND |
| 26.204 | 41 | C29 | 6.69±7.19 | 16.07±21.11 | 13.10±14.94 | 11.78±16.98 | 7.81±6.70 | 6.69±5.27 | 9.76±11.53 | 5.36±7.93 | 5.05±9.54 | 13.03±18.11 | 30.31±22.99 | 17.13±20.57 | 34.03±41.22 | 5.23±5.13 |
| 26.872 | 42 | 11+13+15-Methyl C29 | 1.00±0.86 | ND | ND | 9.37±8.23 | 3.12±2.01 | 1.00±0.00 | ND | 4.63±5.37 | 2.07±2.09 | 4.00±2.51 | 4.07±3.84 | ND | ND | 0.82±1.16 |
| 27.029 | 43 | 7-Methyl C29 | ND | ND | ND | 3.10±2.26 | ND | ND | ND | ND | ND | 0.93±0.65 | 1.10±1.47 | ND | ND | 0.74±1.38 |
| 27.225 | 44 | 5-Methyl C29 | ND | ND | ND | 1.03±0.83 | ND | ND | ND | ND | ND | ND | 0.87±1.52 | ND | ND | 0.32±0.80 |
| 27.477 | 45 | 9,13-DiMethyl C29 | ND | ND | ND | 2.38±2.23 | ND | ND | ND | ND | ND | ND | 1.10±0.81 | ND | 6.98±9.93 | ND |
| 27.697 | 46 | 3-Methyl C29 | 1.34±1.55 | 3.51±3.13 | ND | 10.62±8.17 | 3.59±2.30 | 1.34±0.00 | 1.54±1.64 | 2.34±3.97 | ND | 3.99±4.40 | 13.10±12.95 | 2.43±2.77 | ND | 1.92±1.52 |
| 27.84 | 47 | 5,x-DiMethyl C29 | ND | ND | ND | 1.93±1.31 | ND | ND | ND | ND | ND | ND | ND | ND | ND | ND |
| 28.299 | 48 | 9,13-DiMethyl C29 | ND | ND | ND | 2.10±1.35 | ND | ND | ND | ND | ND | ND | ND | ND | ND | ND |
| 28.755 | 49 | C30 | ND | ND | ND | 0.59±0.40 | ND | ND | ND | ND | ND | ND | 0.56±0.70 | ND | ND | ND |
| 30.647 | 50 | 3,11-DiMethyl C29 | ND | ND | ND | ND | ND | ND | ND | ND | ND | ND | 0.82±0.72 | ND | ND | ND |
| 31.04 | 51 | 2-Methyl C30 | ND | ND | ND | ND | ND | ND | ND | ND | ND | ND | 0.54±0.87 | ND | 1.40±1.96 | ND |
| 29.206 | 52 | C31:1 | ND | 6.68±9.17 | ND | ND | ND | ND | ND | ND | ND | ND | ND | ND | ND | ND |
| 29.495 | 53 | C31 | ND | 3.12±4.20 | ND | ND | ND | ND | ND | ND | ND | 0.74±1.32 | 1.64±2.05 | 1.15±1.57 | ND | ND |
| 29.783 | 54 | 11+13-Methyl C31 | ND | ND | ND | 0.82±1.09 | ND | ND | ND | ND | ND | ND | 0.53±0.80 | ND | ND | ND |
| 31.698 | 55 | 9-Methyl C31 | ND | 0.93±0.65 | ND | ND | ND | ND | ND | ND | ND | ND | ND | ND | ND | ND |
| 31.789 | 56 | 3-Methyl C31 | ND | 0.89±0.93 | ND | ND | ND | ND | ND | ND | ND | ND | ND | ND | ND | ND |
| 30.641 | 57 | C33:2 | ND | 3.43±3.49 | ND | ND | ND | ND | ND | ND | ND | ND | ND | ND | ND | ND |
| 30.69 | 58 | C33:2 | ND | ND | ND | ND | ND | ND | ND | ND | ND | ND | 0.48±0.89 | ND | ND | ND |
| 30.768 | 59 | C33:1 | ND | ND | ND | ND | ND | ND | ND | ND | ND | ND | 0.63±1.09 | ND | ND | ND |
| 31.819 | 60 | C35:2 | ND | 3.89±2.19 | ND | ND | ND | ND | ND | ND | ND | ND | ND | ND | ND | ND |
| 32.512 | 61 | Unknown HC | ND | 2.06±3.17 | ND | ND | ND | ND | ND | ND | ND | ND | ND | ND | ND | ND |

*Tentative identification based on Kovats Index values and match with NIST08 Library database

**Double bond position not assigned

ND = Not Detected

Table S2: List of the compounds extracted from female *Sarcophaga* flies with the total percentage of each compound present, ± the percentage standard deviation for each species (ND = Not Detected).

|  |  |  | ***S.africa*** | ***S.agnata*** | ***S. argyrostoma*** | ***S. carnaria*** | ***S.melanura*** | ***S. pumila*** | ***S. subvicina*** | ***S. sp (carnaria gr)*** | ***S. teretirostris*** | ***S. vagans*** | ***S. variegata*** |
| --- | --- | --- | --- | --- | --- | --- | --- | --- | --- | --- | --- | --- | --- |
|  |  |  | ***n=10*** | ***n=9*** | ***n=10*** | ***n=3*** | ***n=8*** | ***n=8*** | ***n=3*** | ***n=10*** | ***n=8*** | ***n=8*** | ***n=2*** |
| **RT** | **Pk No.** | **Pk ID** | % | % | % | % | % | % | % | % | % | % | % |
| 11.215 | 1 | Aldehyde | ND | 7.99±7.03 | ND | ND | ND | ND | ND | ND | ND | ND | ND |
| 13.08 | 2 | 3-Methyl C21 | ND | ND | ND | ND | ND | 5.94±10.73 | ND | ND | ND | ND | ND |
| 13.259 | 3 | C22 | ND | ND | ND | ND | ND | ND | 0.88±1.03 | 0.08±0.19 | 0.87±0.34 | 1.45±0.70 | 1.06±0.13 |
| 13.676 | 4 | Aldehyde | ND | 1.76±1.42 | ND | ND | ND | ND | ND | ND | ND | ND | ND |
| 14.681 | 5 | 2-Methyl C22 | ND | ND | ND | ND | ND | 3.18±4.46 | ND | ND | ND | ND | ND |
| 14.908 | 6 | C23 | ND | ND | ND | ND | ND | ND | ND | 0.03±0.10 | ND | ND | ND |
| 15.115 | 7 | TriMe C22* | ND | ND | ND | ND | ND | 7.83±10.99 | ND | ND | ND | ND | ND |
| 15.242 | 8 | TriMe C22* | ND | ND | ND | ND | ND | 2.26±3.04 | ND | ND | ND | ND | ND |
| 15.341 | 9 | TriMe C22* | ND | ND | ND | ND | ND | 1.80±2.31 | ND | ND | ND | ND | ND |
| 15.463 | 10 | TriMe C22* | ND | ND | ND | ND | ND | 1.44±2.01 | ND | 0.17±0.61 | ND | ND | ND |
| 15.602 | 11 | 2-Methyl C22 | ND | ND | ND | ND | ND | 1.47±2.03 | ND | 0.36±0.70 | ND | ND | ND |
| 15.704 | 12 | 5,x-DiMethyl C221 | ND | ND | ND | ND | ND | ND | ND | 0.08±0.28 | ND | ND | ND |
| 16.082 | 13 | 3-Methyl C23 | ND | ND | ND | ND | ND | 3.42±4.20 | ND | 0.38±0.79 | ND | ND | ND |
| 16.541 | 14 | C24 | ND | ND | ND | ND | ND | ND | 0.80±1.00 | 0.10±0.27 | ND | ND | 0.97±0.97 |
| 17.156 | 15 | Unknown HC | ND | ND | ND | ND | ND | ND | ND | 0.15±0.41 | ND | ND | ND |
| 17.753 | 16 | C25:1 | ND | ND | ND | ND | ND | ND | ND | 0.17±0.35 | ND | ND | ND |
| 18.027 | 17 | 4-Methyl C24 | ND | ND | ND | ND | ND | 3.03±3.61 | ND | ND | ND | ND | ND |
| 18.358 | 18 | C25 | 22.89±23.16 | 7.20±5.80 | 20.57±18.35 | 2.97±2.93 | 5.99±6.19 | 3.09±3.24 | 4.40±3.29 | 6.06±2.87 | 5.71±8.00 | 5.50±4.51 | 3.35±4.75 |
| 18.518 | 19 | TriMethyl C24* | ND | ND | ND | ND | ND | 9.65±8.16 | ND | ND | ND | ND | ND |
| 19.015 | 20 | 11+13-Methyl C25 | 12.74±11.92 | 3.62±3.50 | 12.07±10.41 | 4.06±2.11 | 2.13±2.34 | 8.14±10.77 | 5.51±7.26 | 9.68±7.78 | 10.19±9.02 | 26.49±34.25 | 6.65±8.80 |
| 19.1 | 21 | 7-Methyl C25 | 3.66±2.79 | ND | ND | ND | ND | ND | 0.47±0.58 | 0.45±0.96 | ND | ND | ND |
| 19.353 | 22 | 5-Methyl C25 | 1.70±1.47 | 1.61±2.07 | ND | ND | 0.89±0.55 | ND | 1.03±1.57 | 0.95±1.30 | ND | 4.62±4.80 | 1.24±2.63 |
| 19.579 | 23 | 11,x-DiMethyl C25* | ND | ND | ND | ND | ND | ND | ND | 0.24±0.50 | ND | 1.85±2.51 | ND |
| 19.741 | 24 | 3-Methyl C25 | 4.17±3.34 | 4.74±5.41 | 5.10±3.42 | 1.28±0.79 | 1.39±1.13 | 7.25±3.94 | 3.85±2.84 | 3.19±2.70 | 5.90±6.58 | 11.02±10.94 | 2.97±4.23 |
| 19.93 | 25 | 5,x-DiMethyl C25* | 1.25±0.97 | ND | ND | ND | ND | ND | 1.29±1.80 | 1.17±1.21 | 1.02±0.52 | 1.60±1.85 | 1.56±2.58 |
| 20.263 | 26 | C26 | 1.30±1.25 | 1.11±0.77 | ND | ND | 0.93±0.86 | ND | 1.31±0.84 | 1.00±0.71 | 1.00±0.45 | 1.87±2.26 | 1.06±0.32 |
| 20.403 | 27 | 3,x-DiMethyl C25* | 1.13±0.84 | ND | ND | ND | ND | 9.59±5.39 | 1.33±1.80 | 0.89±0.94 | 3.77±2.66 | ND | 1.61±2.39 |
| 20.91 | 28 | 12,14-Methyl C26 | 0.65±0.73 | ND | ND | ND | ND | ND | 0.29±0.46 | 0.79±1.13 | ND | 2.12±2.96 | ND |
| 21.006 | 29 | DiMethyl C26* | ND | ND | ND | ND | ND | ND | ND | 0.06±0.21 | ND | ND | ND |
| 21.272 | 30 | C27:2 | ND | ND | ND | ND | 1.13±1.02 | ND | ND | ND | ND | ND | ND |
| 21.404 | 31 | 2-Methyl C26 | ND | ND | ND | ND | ND | ND | ND | 0.10±0.25 | ND | ND | ND |
| 21.546 | 32 | C27:1 | ND | 16.52±20.23 | ND | ND | 3.69±2.62 | ND | ND | ND | 0.86±0.83 | ND | ND |
| 21.76 | 33 | C27:1 | 1.40±1.24 | 3.11±3.26 | ND | ND | 10.27±7.45 | ND | ND | ND | 0.99±1.25 | ND | ND |
| 21.813 | 34 | 2,x-DiMethyl C26* | ND | ND | ND | ND | ND | 2.05±3.04 | ND | ND | ND | ND | ND |
| 22.187 | 35 | C27 | 29.67±32.74 | 30.56±28.29 | 31.95±27.92 | 34.62±42.89 | 35.64±40.12 | 7.24±2.26 | 24.42±19.39 | 29.33±21.73 | 27.17±30.28 | 8.94±4.41 | 23.13±0.42 |
| 22.341 | 36 | TriMethyl* | ND | ND | ND | ND | ND | 8.30±9.51 | ND | ND | ND | ND | ND |
| 22.872 | 37 | 11+13-Methyl C27 | ND | 17.10±18.18 | 6.13±10.23 | 5.80±1.99 | 2.94±1.94 | 5.56±2.56 | 24.27±28.15 | 13.68±16.27 | 18.18±13.81 | 23.89±21.85 | 25.98±41.82 |
| 22.948 | 38 | 9-Methyl C27 | ND | ND | 3.73±3.77 | ND | 1.62±0.84 | ND | ND | ND | ND | ND | ND |
| 23.001 | 39 | 7-Methyl C27 | 1.30±1.08 | ND | ND | 1.05±0.29 | ND | ND | 3.20±2.10 | 1.38±1.90 | ND | ND | 2.43±2.34 |
| 23.219 | 40 | 5-Methyl C27 | 0.36±0.27 | ND | ND | 0.82±0.13 | ND | ND | 1.26±1.65 | 0.44±0.75 | ND | ND | 1.52±1.94 |
| 23.515 | 41 | Unknown HC | 0.92±0.76 | ND | ND | 0.84±0.01 | ND | ND | 2.14±1.84 | 0.36±0.70 | ND | ND | 1.84±2.47 |
| 23.68 | 42 | 3-Methyl C27 | 5.21±4.28 | ND | 7.82±8.04 | 6.60±2.40 | 5.44±3.62 | 3.72±1.55 | 11.62±10.63 | 4.90±5.74 | 7.59±5.05 | 6.03±4.85 | 11.52±10.63 |
| 23.852 | 43 | Unknown HC | ND | ND | ND | 1.74±0.36 | ND | 0.45±0.51 | 1.06±1.75 | 0.29±0.53 | 1.12±1.55 | ND | 1.28±3.25 |
| 24.197 | 44 | C28 | ND | ND | ND | 3.75±1.48 | ND | ND | ND | 1.01±1.17 | 0.75±0.48 | ND | ND |
| 24.36 | 45 | Unknown HC | 1.43±1.11 | ND | ND | ND | ND | 0.42±0.47 | 1.24±1.56 | 0.48±1.18 | 2.17±2.18 | 0.87±1.26 | 1.50±1.49 |
| 24.831 | 46 | 12,14-Methyl C28 | ND | ND | ND | ND | ND | ND | 0.88±1.35 | 0.36±0.67 | ND | ND | 1.06±2.30 |
| 25.167 | 47 | C29:2 | ND | ND | ND | ND | 2.43±3.32 | ND | ND | ND | ND | ND | ND |
| 25.307 | 48 | C29:2 | ND | ND | ND | ND | 0.86±0.79 | ND | ND | ND | ND | ND | ND |
| 25.445 | 49 | C29:1 | ND | ND | ND | ND | 1.96±1.76 | ND | ND | ND | ND | ND | ND |
| 25.576 | 50 | C29:1 | ND | ND | ND | ND | 1.95±1.45 | ND | ND | ND | ND | ND | ND |
| 25.817 | 51 | C29:1 | ND | ND | ND | ND | 2.33±1.77 | ND | ND | ND | ND | ND | ND |
| 26.228 | 52 | C29 | 5.74±6.91 | 2.33±2.07 | 8.39±8.29 | 20.36±28.23 | 8.84±11.39 | 4.10±5.23 | 4.22±3.31 | 9.99±12.11 | 8.11±11.42 | 1.56±0.89 | 3.86±0.99 |
| 26.893 | 53 | 11+13-Methyl C29 | 2.32±2.43 | 1.65±1.53 | 4.21±9.56 | 3.68±3.58 | 3.90±3.24 | ND | 2.79±3.83 | 3.19±3.53 | 2.25±2.87 | 2.16±1.96 | 3.37±5.27 |
| 27.026 | 54 | 7-Methyl C29 | ND | ND | ND | ND | ND | ND | ND | 0.87±0.89 | ND | ND | ND |
| 27.471 | 55 | 9,13-DiMethyl C29 | ND | ND | ND | ND | ND | ND | ND | 0.29±0.69 | ND | ND | ND |
| 27.727 | 56 | 3-Methyl C29 | 2.15±2.72 | 0.69±0.44 | ND | 12.41±12.81 | 5.66±7.60 | ND | 1.70±1.98 | 6.87±6.94 | 2.33±2.71 | ND | 2.05±0.28 |
| 28.291 | 57 | 9,13-DiMethyl C29 | ND | ND | ND | ND | ND | ND | ND | 0.26±0.59 | ND | ND | ND |
| 29.476 | 58 | C31 | ND | ND | ND | ND | ND | ND | ND | 0.15±0.36 | ND | ND | ND |

*Tentative identification based on Kovats Index values and match with NIST08 Library database

**Double bond position not assigned

ND = Not Detected

Table S3: Compounds used for the principal component analysis (PCA), leave-one-out cross validation (LOOCV) and support vector machine (SVM) classification.

| **Pk ID** | 2018 Samples only | Recent and historical specimens of three species | All males | All females | All males and females combined |
| --- | --- | --- | --- | --- | --- |
| Aldehyde |  |  |  | **×** | **×** |
| 3-Methyl C21 |  |  |  | **×** | **×** |
| C22 |  |  |  | **×** | **×** |
| Aldehyde |  |  |  | **×** | **×** |
| 2-Methyl C22 |  |  |  | **×** | **×** |
| C23 | **×** | **×** | **×** |  |  |
| TriMe C22* |  |  |  | **×** | **×** |
| TriMe C22* |  |  |  | **×** | **×** |
| TriMe C22* |  |  |  | **×** | **×** |
| TriMe C22* |  |  |  | **×** |  |
| 2-Methyl C22 |  |  |  | **×** |  |
| 5,x-DiMethyl C22* |  |  |  |  |  |
| 13+15-Methyl C23 | **×** |  | **×** |  | **×** |
| 9+11-Methyl C23 |  |  | **×** |  | **×** |
| 3-Methyl C23 | **×** |  |  | **×** | **×** |
| C24 |  |  |  | **×** |  |
| Unknown HC |  |  |  |  |  |
| 11+13-Methyl C24 |  |  |  |  |  |
| 9+11+13-Methyl C24 |  |  |  |  |  |
| C25:1** |  |  |  | **×** |  |
| 4-Methyl C24 |  |  |  | **×** | **×** |
| C25 |  | **×** |  | **×** |  |
| TriMethyl C24* |  |  |  | **×** |  |
| 11+13-Methyl C25 |  | **×** |  | **×** |  |
| 9-Methyl C25 |  |  |  |  |  |
| 7-Methyl C25 |  |  |  | **×** |  |
| 5-Methyl C25 |  | **×** | **×** | **×** | **×** |
| 11,x-DiMethyl C25* |  | **×** | **×** | **×** |  |
| 3-Methyl C25 |  | **×** | **×** | **×** |  |
| 5,x-DiMethyl C25* |  | **×** |  | **×** |  |
| C26 |  | **×** |  | **×** |  |
| 3,x-DiMethyl C25* |  | **×** |  | **×** |  |
| 12,14Methyl C26 |  | **×** | **×** | **×** | **×** |
| DiMethyl C26* |  |  |  |  |  |
| C27:2** |  |  | **×** | **×** | **×** |
| 2-Methyl C26 |  |  |  |  | **×** |
| C27:1** |  |  | **×** | **×** | **×** |
| C27:1** |  |  | **×** | **×** | **×** |
| 2,x-DiMethyl C26* |  |  |  | **×** | **×** |
| C27 | **×** | **×** | **×** | **×** | **×** |
| TriMethyl* |  |  |  | **×** | **×** |
| 11+13-Methyl C27 | **×** | **×** | **×** | **×** | **×** |
| 9-Methyl C27 |  |  |  | **×** | **×** |
| 7-Methyl C27 | **×** | **×** | **×** | **×** | **×** |
| 5-Methyl C27 |  | **×** | **×** | **×** | **×** |
| Unknown HC | **×** | **×** | **×** | **×** | **×** |
| 3-Methyl C27 | **×** |  | **×** | **×** | **×** |
| Unknown HC |  | **×** | **×** | **×** | **×** |
| C28 | **×** | **×** | **×** | **×** | **×** |
| Unknown HC |  | **×** | **×** | **×** | **×** |
| 2-Methyl C28 | **×** | **×** | **×** |  | **×** |
| 12,14-Methyl C28 |  |  |  | **×** |  |
| C29:2** |  |  |  | **×** |  |
| C29:2** |  |  |  | **×** |  |
| C29:2** |  |  |  |  |  |
| C29:1** |  |  |  | **×** |  |
| C29:1** |  |  |  | **×** |  |
| Unknown HC |  |  |  |  |  |
| C29:1** |  |  |  | **×** |  |
| C29 |  |  |  | **×** |  |
| 11+13+15-Methyl C29 | |  |  |  |  |
| 7-Methyl C29 |  |  |  | **×** |  |
| 5-Methyl C29 |  |  |  |  |  |
| 9,13-DiMethyl C29* |  |  |  |  |  |
| 3-Methyl C29 |  |  |  | **×** |  |
| 5,x-DiMethyl C29* |  |  |  |  |  |
| 9,13-DiMethyl C29* |  |  |  |  |  |
| C30 |  |  |  |  |  |
| 3,11-DiMethyl C29* | **×** |  | **×** |  | **×** |
| 2-Methyl C30 | **×** | **×** | **×** |  | **×** |
| C31:1** |  |  |  |  |  |
| C31 |  |  |  |  |  |
| 11+13-Methyl C31 |  |  |  |  |  |
| 9-Methyl C31 |  |  |  |  |  |
| 3-Methyl C31 |  |  | **×** |  |  |
| C33:2** |  |  | **×** |  | **×** |
| C33:2** |  |  |  |  | **×** |
| C33:1** |  |  |  |  |  |
| C35:2** |  |  | **×** |  |  |
| Unknown HC |  |  | **×** |  |  |

*Tentative identification based on Kovats Index values and match with NIST08 library database

**Double bond position not assigned

Table S4: Factor Loadings for compounds used to calculate PCA in Figure 2.

| **Retention time** | **Compound** | **Component 1** | **Component 2** | **Component 3** |
| --- | --- | --- | --- | --- |
| 17.981 | C23 | 100 | 9.85 | 25.62 |
| 18.799 | 13+15MeC23 | 73.11 | 21.75 | 131.86 |
| 19.45 | 3MeC23 | 72.46 | 21.6 | 134.27 |
| 22.19 | C27 | 38.72 | 139.46 | 28.8 |
| 22.865 | 11+13-Me C27 | 56.09 | 103.76 | 10.6 |
| 23.037 | 7-MeC27 | 49.94 | 24.76 | 66.57 |
| 23.505 | Unknown HC | 67.51 | 107.32 | 1.15 |
| 23.665 | 3-MeC27 | 50.6 | 17.87 | 43.44 |
| 24.215 | C28 | 42.18 | 86.49 | 90.59 |
| 29.324 | 2MeC28 | 80.51 | 3.26 | 89.2 |
| 30.647 | 3,11diMeC29 | 3.67 | 151.96 | 41.13 |
| 31.04 | 2MeC30 | 75.86 | 22.58 | 47.43 |

Table S5: Factor Loadings for compounds used to calculate PCA in Figure 3.

| **Retention time** | **Compound** | **Component 1** | **Component 2** | **Component 3** |
| --- | --- | --- | --- | --- |
| 17.981 | C23 | 15.33 | 6.86 | 67.21 |
| 18.333 | C25 | 1.33 | 92.71 | 32.33 |
| 19.006 | 11+13-Me C25 | 51.68 | 42.32 | 0.11 |
| 19.316 | 5-Me C25 | 12.44 | 95.24 | 39.04 |
| 19.5 | 11,XDiMeC25 | 33.43 | 48.09 | 3.95 |
| 19.736 | 3-Me C25 | 62.18 | 58.54 | 0.08 |
| 19.9 | 5,XdiMeC25 | 76.03 | 2.63 | 1.45 |
| 20.251 | C26 | 1.96 | 55.62 | 52.85 |
| 20.398 | 3,XDiMeC25 | 71.24 | 5.22 | 1.83 |
| 20.948 | 12,14MeC26 | 34.22 | 45.25 | 0.61 |
| 22.19 | C27 | 49.62 | 4.65 | 127.32 |
| 22.865 | 11+13-Me C27 | 9.36 | 16.06 | 133.7 |
| 23.037 | 7-MeC27 | 100 | 0.16 | 5.3 |
| 23.235 | 5-MeC27 | 38.95 | 98.16 | 1.4 |
| 23.505 | Unknown HC | 4.59 | 8.74 | 213.98 |
| 23.665 | 3-MeC27 | 40.73 | 73.26 | 1.19 |
| 23.879 | Unknown HC | 40.08 | 76.46 | 1.08 |
| 24.215 | C28 | 74.57 | 19.1 | 0.22 |
| 24.383 | Unknown HC | 32 | 91.87 | 0.8 |
| 29.324 | 2MeC28 | 58.68 | 0.73 | 38.26 |
| 31.04 | 2MeC30 | 34.24 | 0.99 | 119.96 |

Table S6: Support Vector Machine (SVM) class assignments for unknows. Shaded species highlight the three misclassified results.

| **File name** | **Classified as** | **Known Class and Gender** |
| --- | --- | --- |
| *S. vegans-167* | *S. vagans F* | *S. vagans F* |
| *S. subvicina-58* | *S. subvicina M* | *S. subvicina M* |
| *S. agnata-142* | *S. agnata F* | *S. agnata F* |
| *S. teretirostris-158* | *S. teretirostris F* | *S. teretirostris F* |
| *S. argyrostoma-152* | *S. argyrostoma F* | *S. argyrostoma F* |
| *S. subvicina-95* | *S. subvicina M* | *S. subvicina M* |
| *S. variegata-66* | *S. variegata M* | *S. variegata M* |
| *S. vegans-165* | *S. vagans F* | *S. vagans F* |
| *S. subvicina-100* | *S. subvicina M* | *S. subvicina M* |
| *S. pumila -179* | *S. pumila F* | *S. pumila F* |
| *S. agnata-134* | *S. agnata F* | *S. agnata F* |
| *S. melanura-7* | *S. melanura M* | *S. melanura M* |
| *S. argyrostoma-76* | *S. argyrostoma M* | *S. argyrostoma M* |
| *S. agnata-141* | *S. agnata F* | *S. agnata F* |
| *S. pumila-33* | *S. pumila M* | *S. pumila M* |
| *S. pumila-34* | *S. pumila M* | *S. pumila M* |
| *S. argyrostoma-74* | *S. argyrostoma M* | *S. argyrostoma M* |
| *S. vagans-163* | *S. teretirostris M* | *S. vagans F* |
| *S. subvicina-52* | *S. subvicina M* | *S. subvicina M* |
| *S. vagans-49* | *S. vagans M* | *S. vagans M* |
| *S. carnaria-73* | *S. carnaria M* | *S. carnaria M* |
| *S. argyrostoma-75* | *S. argyrostoma M* | *S. argyrostoma M* |
| *S. carnaria-83* | *S. carnaria M* | *S. carnaria M* |
| *S. agnata-139* | *S. agnata F* | *S. agnata F* |
| *S. pumila -185* | *S. pumila F* | *S. pumila F* |
| *S. africa -129* | *S. africa F* | *S. africa F* |
| *S. africa-14* | *S. africa M* | *S. africa M* |
| *S. melanura-121* | *S. melanura F* | *S. melanura F* |
| *S. variegata-107* | *S. variegata M* | *S. variegata F* |
| *S. africa -130* | *S. africa F* | *S. africa F* |
| *S. vagans-45* | *S. vagans M* | *S. vagans M* |
| *S. argyrostoma-145* | *S. argyrostoma F* | *S. argyrostoma F* |
| *S. melanura-1* | *S. melanura M* | *S. melanura M* |
| *S. pumila -180* | *S. pumila F* | *S. pumila F* |
| *S. melanura-6* | *S. melanura M* | *S. melanura M* |
| *S. argyrostoma-150* | *S. argyrostoma F* | *S. argyrostoma F* |
| *S. subvicina-97* | *S. subvicina M* | *S. subvicina M* |
| *S. variegata-106* | *S. variegata M* | *S. variegata F* |
| *S. teretirostris-155* | *S. teretirostris F* | *S. teretirostris F* |
| *S. melanura-0* | *S. melanura M* | *S. melanura M* |
| *S. argyrostoma-144* | *S. argyrostoma F* | *S. argyrostoma F* |
| *S. melanura-117* | *S. melanura F* | *S. melanura F* |
| *S. argyrostoma-78* | *S. argyrostoma M* | *S. argyrostoma M* |
| *S. africa -128* | *S. africa F* | *S. africa F* |
| *S. melanura-3* | *S. melanura M* | *S. melanura M* |
| *S. variegata-60* | *S. variegata M* | *S. variegata M* |
| *S. teretirostris-160* | *S. teretirostris F* | *S. teretirostris F* |
| *S. agnata-23* | *S. agnata M* | *S. agnata M* |
| *S. carnaria-87* | *S. carnaria M* | *S. carnaria M* |
| *S. vagans-48* | *S. vagans M* | *S. vagans M* |

Table S7: Year and locality of collection for analysed male *Sarcophaga* specimens.

| **Specimen no.** | **Unique identifier (NHMUK)** | **Species (Male)** | **Year** | **Locality** |
| --- | --- | --- | --- | --- |
| 1 | 010397091 | *Sarcophaga* (Bercaea) *africa* | 1953 | London |
| 2 | 010397093 | *Sarcophaga* (*Bercaea*) *africa* | 1953 | London |
| 3 | 010397094 | *Sarcophaga* (*Bercaea*) *africa* | 1953 | London |
| 4 | 010397095 | *Sarcophaga* (*Bercaea*) *africa* | 1953 | London |
| 5 | 010397096 | *Sarcophaga* (*Bercaea*) *africa* | 1953 | London |
| 6 | 010397097 | *Sarcophaga* (*Bercaea*) *africa* | 1953 | London |
| 7 | 010397098 | *Sarcophaga* (*Bercaea*) *africa* | 1953 | London |
| 8 | 010397099 | *Sarcophaga* (*Bercaea*) *africa* | 1953 | London |
| 9 | 010397100 | *Sarcophaga* (*Bercaea*) *africa* | 1954 | London |
| 10 | 010397227 | *Sarcophaga* (*Helicophagella*) *agnata* | 1932 | Wyre Forest |
| 11 | 010397228 | *Sarcophaga* (*Helicophagella*) *agnata* | 1941 | Lynton |
| 12 | 010397229 | *Sarcophaga* (*Helicophagella*) *agnata* | 1941 | Lynton |
| 13 | 010397231 | *Sarcophaga* (*Helicophagella*) *agnata* | 1941 | Lynton |
| 14 | 010397232 | *Sarcophaga* (*Helicophagella*) *agnata* | 1941 | Lynton |
| 15 | 010397233 | *Sarcophaga* (*Helicophagella*) *agnata* | 1937 | Kent |
| 16 | 010397234 | *Sarcophaga* (*Helicophagella*) *agnata* | 1939 | Lancashire |
| 17 | 010397235 | *Sarcophaga* (*Helicophagella*) *agnata* | 1941 | Woodditton |
| 18 | 010397236 | *Sarcophaga* (*Helicophagella*) *agnata* | 1935 | Hell Coppice |
| 19 | 010397340 | *Sarcophaga* (*Heteronychia*) *pumila* | 1949 | Edington |
| 20 | 010397341 | *Sarcophaga* (*Heteronychia*) *pumila* | 1949 | Edington |
| 21 | 010397342 | *Sarcophaga* (*Heteronychia*) *pumila* | 1949 | Edington |
| 22 | 010397344 | *Sarcophaga* (*Heteronychia*) *pumila* | 1949 | Edington |
| 23 | 010397345 | *Sarcophaga* (*Heteronychia*) *pumila* | 1949 | Edington |
| 24 | 010397346 | *Sarcophaga* (*Heteronychia*) *pumila* | 1963 | Swinbrook |
| 25 | 010397347 | *Sarcophaga* (*Heteronychia*) *pumila* | 1957 | Oxwich |
| 26 | 010397348 | *Sarcophaga* (*Heteronychia*) *pumila* | 1953 | Oxwich |
| 27 | 010397360 | *Sarcophaga* (*Helicophagella*) *melanura* | 1977 | Barry Links, Angus |
| 28 | 010397361 | *Sarcophaga* (*Helicophagella*) *melanura* | 1977 | Barry Links, Angus |
| 29 | 010397362 | *Sarcophaga* (*Helicophagella*) *melanura* | 1952 | Portishead |
| 30 | 010397363 | *Sarcophaga* (*Helicophagella*) *melanura* | 1953 | Berrow |
| 31 | 010397364 | *Sarcophaga* (*Helicophagella*) *melanura* | 1953 | Oxwich |
| 32 | 010397365 | *Sarcophaga* (*Helicophagella*) *melanura* | 1949 | Filton |
| 33 | 010397366 | *Sarcophaga* (*Helicophagella*) *melanura* | 1953 | Oxwich |
| 34 | 010397367 | *Sarcophaga* (*Helicophagella*) *melanura* | 2013 | Isles of Scilly |
| 35 | 010579714 | *Sarcophaga* (*Helicophagella*) *melanura* | 2013 | Isles of Scilly |
| 36 | 010579715 | *Sarcophaga* (*Helicophagella*) *melanura* | 2013 | Isles of Scilly |
| 37 | 010397376 | *Sarcophaga* (*Liopygia*) *argyrostoma* | 1953 | London |
| 38 | 010397377 | *Sarcophaga* (*Liopygia*) *argyrostoma* | 1952 | London |
| 39 | 010397378 | *Sarcophaga* (*Liopygia*) *argyrostoma* | 1952 | London |
| 40 | 010397380 | *Sarcophaga* (*Liopygia*) *argyrostoma* | 1953 | London |
| 41 | 010397381 | *Sarcophaga* (*Liopygia*) *argyrostoma* | 1952 | London |
| 42 | 010397382 | *Sarcophaga* (*Liopygia*) *argyrostoma* | 1953 | London |
| 43 | 010397383 | *Sarcophaga* (*Liopygia*) *argyrostoma* | 1959 | Bristol |
| 44 | 010397384 | *Sarcophaga* (*Liopygia*) *argyrostoma* | 1953 | Portishead |
| 45 | 010397385 | *Sarcophaga* (*Liopygia*) *argyrostoma* | 1969 | Bristol |
| 46 | 010397396 | *Sarcophaga* (*Liosarcophaga*) *teretirostris* | 1954 | Hadleigh |
| 47 | 010397397 | *Sarcophaga* (*Liosarcophaga*) *teretirostris* | 1932 | Carbis Bay |
| 48 | 010397398 | *Sarcophaga* (*Liosarcophaga*) *teretirostris* | 1949 | Coombe Dingle |
| 49 | 010397399 | *Sarcophaga* (*Liosarcophaga*) *teretirostris* | 1977 | Hartslock Hill |
| 50 | 010397401 | *Sarcophaga* (*Liosarcophaga*) *teretirostris* | 1953 | Seaton |
| 51 | 010397416 | *Sarcophaga* (*Heteronychia*) *vagans* | 1924 | Cirencester |
| 52 | 010397417 | *Sarcophaga* (*Heteronychia*) *vagans* | 1934 | Cornwall |
| 53 | 010397418 | *Sarcophaga* (*Heteronychia*) *vagans* | 1960 | Boxhill, Surrey |
| 54 | 010397419 | *Sarcophaga* (*Heteronychia*) *vagans* | 1954 | New Forest |
| 55 | 010397420 | *Sarcophaga* (*Heteronychia*) *vagans* | 1934 | Swanage |
| 56 | 010397422 | *Sarcophaga* (*Heteronychia*) *vagans* | 1948 | Walton Moor |
| 57 | 010397423 | *Sarcophaga* (*Heteronychia*) *vagans* | 1947 | Filton |
| 58 | 010397424 | *Sarcophaga* (*Heteronychia*) *vagans* | 1949 | Coombe Dingle |
| 59 | 010397425 | *Sarcophaga* (*Heteronychia*) *vagans* | 1949 | Sharpham |
| 60 | 010392546 | *Sarcophaga* (*Sarcophaga*) *carnaria* | 1952 | London |
| 61 | 010392547 | *Sarcophaga* (*Sarcophaga*) *carnaria* | 1952 | London |
| 62 | 010392639 | *Sarcophaga* (*Sarcophaga*) *carnaria* | 1983 | Bristol |
| 63 | 010392640 | *Sarcophaga* (*Sarcophaga*) *carnaria* | 1983 | Bristol |
| 64 | 010241253 | *Sarcophaga* (*Sarcophaga*) *carnaria* | 1931 | Harewood Forest |
| 65 | 010393131 | *Sarcophaga* (*Sarcophaga*) *subvicina* | 1913 | Cusop Dingle |
| 66 | 010393134 | *Sarcophaga* (*Sarcophaga*) *subvicina* | 1901 | Wyre Forest |
| 67 | 010393136 | *Sarcophaga* (*Sarcophaga*) *subvicina* | 1954 | New Forest |
| 68 | 010393166 | *Sarcophaga* (*Sarcophaga*) *subvicina* | 1954 | New Forest |
| 69 | 010393167 | *Sarcophaga* (*Sarcophaga*) *subvicina* | 1949 | River Tamar, Devon |
| 70 | 010393261 | *Sarcophaga* (*Sarcophaga*) *subvicina* | 1983 | Bristol |
| 71 | 010393263 | *Sarcophaga* (*Sarcophaga*) *subvicina* | 2013 | Isles of Scilly |
| 72 | 010393403 | *Sarcophaga* (*Sarcophaga*) *subvicina* | 2013 | Isles of Scilly |
| 73 | 010393404 | *Sarcophaga* (*Sarcophaga*) *subvicina* | 2013 | Isles of Scilly |
| 74 | 010393405 | *Sarcophaga* (*Sarcophaga*) *subvicina* | 2013 | Isles of Scilly |
| 75 | 010241317 | *Sarcophaga* (*Sarcophaga*) *variegata* | 1913 | Cusop Dingle |
| 76 | 010392574 | *Sarcophaga* (*Sarcophaga*) *variegata* | 1927 | Isles of Scilly |
| 77 | 010393694 | *Sarcophaga* (*Sarcophaga*) *variegata* | 1953 | Portsdown |
| 78 | 010393696 | *Sarcophaga* (*Sarcophaga*) *variegata* | 1953 | Seaton |
| 79 | 010393849 | *Sarcophaga* (*Sarcophaga*) *variegata* | 2013 | Porton Down |
| 80 | 010393858 | *Sarcophaga* (*Sarcophaga*) *variegata* | 2013 | Isles of Scilly |
| 81 | 010393859 | *Sarcophaga* (*Sarcophaga*) *variegata* | 2013 | Isles of Scilly |
| 82 | 010393844 | *Sarcophaga* (*Sarcophaga*) *variegata* | 1977 | Stormont Loch, Perth |
| 83 | 010393845 | *Sarcophaga* (*Sarcophaga*) *variegata* | 1977 | Stormont Loch, Perth |
| 84 | 013433240 | *Sarcophaga* (*Sarcophaga*) *variegata* | 2018 | Salisbury Plain |
| 85 | 013433241 | *Sarcophaga* (*Sarcophaga*) *variegata* | 2018 | Salisbury Plain |
| 86 | 013433242 | *Sarcophaga* (*Sarcophaga*) *variegata* | 2018 | Salisbury Plain |
| 87 | 013433243 | *Sarcophaga* (*Sarcophaga*) *variegata* | 2018 | Salisbury Plain |
| 88 | 013433244 | *Sarcophaga* (*Sarcophaga*) *variegata* | 2018 | Salisbury Plain |
| 89 | 013433245 | *Sarcophaga* (*Sarcophaga*) *variegata* | 2018 | Salisbury Plain |
| 90 | 013433246 | *Sarcophaga* (*Sarcophaga*) *variegata* | 2018 | Salisbury Plain |
| 91 | 013433247 | *Sarcophaga* (*Sarcophaga*) *variegata* | 2018 | Salisbury Plain |
| 92 | N/A | *Sarcophaga* (*Sarcophaga*) *variegata* | 2018 | Harmondsworth, London |
| 93 | N/A | *Sarcophaga* (*Sarcophaga*) *variegata* | 2018 | Harmondsworth, London |
| 94 | N/A | *Sarcophaga* (*Sarcophaga*) *subvicina* | 2018 | Harmondsworth, London |
| 95 | N/A | *Sarcophaga* (*Sarcophaga*) *subvicina* | 2018 | Harmondsworth, London |
| 96 | N/A | *Sarcophaga* (*Sarcophaga*) *subvicina* | 2018 | Harmondsworth, London |
| 97 | N/A | *Sarcophaga* (*Sarcophaga*) *subvicina* | 2018 | Harmondsworth, London |
| 98 | N/A | *Sarcophaga* (*Sarcophaga*) *subvicina* | 2018 | Harmondsworth, London |
| 99 | N/A | *Sarcophaga* (*Sarcophaga*) *subvicina* | 2018 | Harmondsworth, London |
| 100 | N/A | *Sarcophaga* (*Sarcophaga*) *subvicina* | 2018 | Harmondsworth, London |
| 101 | N/A | *Sarcophaga* (*Sarcophaga*) *subvicina* | 2018 | Harmondsworth, London |
| 102 | N/A | *Sarcophaga* (*Sarcophaga*) *subvicina* | 2018 | Harmondsworth, London |
| 103 | 013433230 | *Sarcophaga* (*Sarcophaga*) *carnaria* | 2018 | Salisbury Plain |
| 104 | 013433231 | *Sarcophaga* (*Sarcophaga*) *carnaria* | 2018 | Salisbury Plain |
| 105 | 013433232 | *Sarcophaga* (*Sarcophaga*) *carnaria* | 2018 | Salisbury Plain |
| 106 | 013433233 | *Sarcophaga* (*Sarcophaga*) *carnaria* | 2018 | Salisbury Plain |
| 107 | 013433234 | *Sarcophaga* (*Sarcophaga*) *carnaria* | 2018 | Salisbury Plain |
| 108 | 013433235 | *Sarcophaga* (*Sarcophaga*) *carnaria* | 2018 | Salisbury Plain |
| 109 | 013433236 | *Sarcophaga* (*Sarcophaga*) *carnaria* | 2018 | Salisbury Plain |
| 110 | 013433237 | *Sarcophaga* (*Sarcophaga*) *carnaria* | 2018 | Salisbury Plain |
| 111 | 013433238 | *Sarcophaga* (*Sarcophaga*) *carnaria* | 2018 | Salisbury Plain |
| 112 | 013433215 | *Sarcophaga* (*Sarcophaga*) *crassipalpis* | 2018 | South Kensington, London |
| 113 | 013433216 | *Sarcophaga* (*Sarcophaga*) *crassipalpis* | 2018 | South Kensington, London |
| 114 | 013433217 | *Sarcophaga* (*Sarcophaga*) *crassipalpis* | 2018 | South Kensington, London |
| 115 | 013433218 | *Sarcophaga* (*Sarcophaga*) *crassipalpis* | 2018 | South Kensington, London |
| 116 | 013433219 | *Sarcophaga* (*Sarcophaga*) *crassipalpis* | 2018 | South Kensington, London |

Table S8: Year and locality of collection for analysed female *Sarcophaga* specimens.

| **Specimen no.** | **Unique identifier (NHMUK)** | **Species (Female)** | **Year collected** | **Locality** |
| --- | --- | --- | --- | --- |
| 1 | 010397172 | *Sarcophaga* (*Bercaea*) *africa* | 1953 | London |
| 2 | 010397173 | *Sarcophaga* (*Bercaea*) *africa* | 1953 | London |
| 3 | 010397174 | *Sarcophaga* (*Bercaea*) *africa* | 1953 | London |
| 4 | 010397175 | *Sarcophaga* (*Bercaea*) *africa* | 1953 | London |
| 5 | 010397176 | *Sarcophaga* (*Bercaea*) *africa* | 1953 | London |
| 6 | 010397177 | *Sarcophaga* (*Bercaea*) *africa* | 1953 | London |
| 7 | 010397178 | *Sarcophaga* (*Bercaea*) *africa* | 1953 | London |
| 8 | 010397179 | *Sarcophaga* (*Bercaea*) *africa* | 1953 | London |
| 9 | 010397180 | *Sarcophaga* (*Bercaea*) *africa* | 1953 | London |
| 10 | 010397181 | *Sarcophaga* (*Bercaea*) *africa* | 1953 | London |
| 11 | 010397296 | *Sarcophaga* (*Helicophagella*) *agnata* | 1933 | Alfrick |
| 12 | 010397297 | *Sarcophaga* (*Helicophagella*) *agnata* | 1941 | Lynton |
| 13 | 010397299 | *Sarcophaga* (*Helicophagella*) *agnata* | 1937 | Shute |
| 14 | 010397300 | *Sarcophaga* (*Helicophagella*) *agnata* | 1937 | Kent |
| 15 | 010397301 | *Sarcophaga* (*Helicophagella*) *agnata* | 1937 | Kent |
| 16 | 010397302 | *Sarcophaga* (*Helicophagella*) *agnata* | 1941 | Lynton |
| 17 | 010397303 | *Sarcophaga* (*Helicophagella*) *agnata* | 1937 | Kent |
| 18 | 010397304 | *Sarcophaga* (*Helicophagella*) *agnata* | 1912 | Bagley Wood, Oxfordshire |
| 19 | 010397305 | *Sarcophaga* (*Helicophagella*) *agnata* | 1928 | St. Ives |
| 20 | 010397350 | *Sarcophaga* (*Heteronychia*) *pumila* | 1983 | Kingsweston |
| 21 | 010397351 | *Sarcophaga* (*Heteronychia*) *pumila* | 1954 | Oxwich |
| 22 | 010397352 | *Sarcophaga* (*Heteronychia*) *pumila* | 1952 | Breamore, Hants |
| 23 | 010397353 | *Sarcophaga* (*Heteronychia*) *pumila* | 1953 | Edington |
| 24 | 010397355 | *Sarcophaga* (*Heteronychia*) *pumila* | 1953 | Babraham |
| 25 | 010397356 | *Sarcophaga* (*Heteronychia*) *pumila* | 1983 | Cricklade |
| 26 | 010397357 | *Sarcophaga* (*Heteronychia*) *pumila* | 1953 | Monkton |
| 27 | 010397358 | *Sarcophaga* (*Heteronychia*) *pumila* | 1955 | Portsdown, Hants |
| 28 | 010579718 | *Sarcophaga* (*Helicophagella*) *melanura* | 1951 | Sandwich Bay |
| 29 | 010397368 | *Sarcophaga* (*Helicophagella*) *melanura* | 1951 | Tal-Y-Bont |
| 30 | 010397369 | *Sarcophaga* (*Helicophagella*) *melanura* | 1954 | Folkestone |
| 31 | 010397371 | *Sarcophaga* (*Helicophagella*) *melanura* | 1953 | Oxwich |
| 32 | 010397372 | *Sarcophaga* (*Helicophagella*) *melanura* | 1953 | St. Margaret, Kent |
| 33 | 010397373 | *Sarcophaga* (*Helicophagella*) *melanura* | 1952 | Oxwich |
| 34 | 010397374 | *Sarcophaga* (*Helicophagella*) *melanura* | 1934 | Holkham |
| 35 | 010397375 | *Sarcophaga* (*Helicophagella*) *melanura* | 1933 | Carbis Bay |
| 36 | 010397386 | *Sarcophaga* (*Liopygia*) *argyrostoma* | 1909 | London |
| 37 | 010397387 | *Sarcophaga* (*Liopygia*) *argyrostoma* | 1952 | London |
| 38 | 010397388 | *Sarcophaga* (*Liopygia*) *argyrostoma* | 1952 | London |
| 39 | 010397389 | *Sarcophaga* (*Liopygia*) *argyrostoma* | 1952 | London |
| 40 | 010397390 | *Sarcophaga* (*Liopygia*) *argyrostoma* | 1953 | London |
| 41 | 010397391 | *Sarcophaga* (*Liopygia*) *argyrostoma* | 1953 | London |
| 42 | 010397392 | *Sarcophaga* (*Liopygia*) *argyrostoma* | 1953 | London |
| 43 | 010397393 | *Sarcophaga* (*Liopygia*) *argyrostoma* | 1969 | Bristol |
| 44 | 010397394 | *Sarcophaga* (*Liopygia*) *argyrostoma* | [none] | [none] |
| 45 | 010397395 | *Sarcophaga* (*Liopygia*) *argyrostoma* | 1936 | London |
| 46 | 010397406 | *Sarcophaga* (*Liosarcophaga*) *teretirostris* | 1944 | Southwell, Portland |
| 47 | 010397407 | *Sarcophaga* (*Liosarcophaga*) *teretirostris* | 1953 | St. Margaret, Kent |
| 48 | 010397408 | *Sarcophaga* (*Liosarcophaga*) *teretirostris* | 1953 | St. Margaret, Kent |
| 49 | 010397409 | *Sarcophaga* (*Liosarcophaga*) *teretirostris* | 1953 | St. Margaret, Kent |
| 50 | 010397410 | *Sarcophaga* (*Liosarcophaga*) *teretirostris* | 1953 | Portishead |
| 51 | 010397411 | *Sarcophaga* (*Liosarcophaga*) *teretirostris* | 1952 | Sandbanks, Dorset |
| 52 | 010397412 | *Sarcophaga* (*Liosarcophaga*) *teretirostris* | 1954 | Portsdown |
| 53 | 010397413 | *Sarcophaga* (*Liosarcophaga*) *teretirostris* | 1953 | Rowlands Castle |
| 54 | 010397426 | *Sarcophaga* (*Heteronychia*) *vagans* | 1935 | Carbis Bay |
| 55 | 010397427 | *Sarcophaga* (*Heteronychia*) *vagans* | 1937 | Carbis Bay |
| 56 | 010397428 | *Sarcophaga* (*Heteronychia*) *vagans* | 1941 | Alfrick |
| 57 | 010397429 | *Sarcophaga* (*Heteronychia*) *vagans* | 1953 | Banstead |
| 58 | 010397430 | *Sarcophaga* (*Heteronychia*) *vagans* | 1954 | Wareham |
| 59 | 010397431 | *Sarcophaga* (*Heteronychia*) *vagans* | 1948 | Filton |
| 60 | 010397434 | *Sarcophaga* (*Heteronychia*) *vagans* | 1941 | Chippenham Fen |
| 61 | 010397435 | *Sarcophaga* (*Heteronychia*) *vagans* | 1953 | Backwell Down |
| 62 | 010241252 | *Sarcophaga* (*Sarcophaga*) *carnaria* | 1935 | Shrawley Wood |
| 63 | 010241254 | *Sarcophaga* (*Sarcophaga*) *carnaria* | 1931 | Harewood Forest |
| 64 | 010241247 | *Sarcophaga* (*Sarcophaga*) *carnaria* | 1909 | New Forest |
| 65 | 010393132 | *Sarcophaga* (*Sarcophaga*) *subvicina* | 1913 | Cusop Dingle |
| 66 | 010393135 | *Sarcophaga* (*Sarcophaga*) *subvicina* | 1913 | Stoke Wood, Herefordshire |
| 67 | 010393137 | *Sarcophaga* (*Sarcophaga*) *subvicina* | 1901 | Wyre Forest |
| 68 | 010241318 | *Sarcophaga* (*Sarcophaga*) *variegata* | 1913 | Cusop Dingle |
| 69 | 010392575 | *Sarcophaga* (*Sarcophaga*) *variegata* | 1927 | Isles of Scilly |

Table S9: Specimens that did not yield a sufficient chemical profile for analysis.

| **Unique identifier (NHMUK).** | **Species** | **Gender** | **Year** | **Locality** |
| --- | --- | --- | --- | --- |
| 010397092 | *Sarcophaga (Bercaea) africa* | Male | 1953 | London |
| 010397230 | *Sarcophaga (Helicophagella) agnata* | Male | 1941 | Lynton |
| 010397343 | *Sarcophaga (Heteronychia) pumila* | Male | 1949 | Edington |
| 010397349 | *Sarcophaga (Heteronychia) pumila* | Male | 1953 | St. Margaret, Kent |
| 010397379 | *Sarcophaga (Liopygia) argyrostoma* | Male | 1953 | London |
| 010397400 | *Sarcophaga (Liosarcophaga) teretirostris* | Male | 1941 | Lynton |
| 010397402 | *Sarcophaga (Liosarcophaga) teretirostris* | Male | 1954 | Portsdown, Hants |
| 010397403 | *Sarcophaga (Liosarcophaga) teretirostris* | Male | 2015 | Salisbury Plain |
| 010397404 | *Sarcophaga (Liosarcophaga) teretirostris* | Male | 2015 | Salisbury Plain |
| 010397405 | *Sarcophaga (Liosarcophaga) teretirostris* | Male | 2015 | Salisbury Plain |
| 010397421 | *Sarcophaga (Heteronychia) vagans* | Male | 1957 | Oxwich |
| 010392652 | *Sarcophaga (Sarcophaga) carnaria* | Male | 2013 | Porton Down |
| 010392655 | *Sarcophaga (Sarcophaga) carnaria* | Male | 2013 | Porton Down |
| 010392656 | *Sarcophaga (Sarcophaga) carnaria* | Male | 2013 | Porton Down |
| 010241251 | *Sarcophaga (Sarcophaga) carnaria* | Male | 1935 | Shrawley Wood |
| 010241246 | *Sarcophaga (Sarcophaga) carnaria* | Male | 1909 | New Forest |
| 010241249 | *Sarcophaga (Sarcophaga) variegata* | Male | 1931 | Aston Rowant |
| N/A | *Sarcophaga (Sarcophaga) subvicina* | Male | 2018 | Harmondsworth, London |
| 013433239 | *Sarcophaga (Sarcophaga) carnaria* | Male | 2018 | Salisbury Plain |
| 013433195 | *Sarcophaga (Sarcophaga) crassipalpis* | Male | 2018 | South Kensington, London |
| 013433196 | *Sarcophaga (Sarcophaga) crassipalpis* | Male | 2018 | South Kensington, London |
| 013433197 | *Sarcophaga (Sarcophaga) crassipalpis* | Male | 2018 | South Kensington, London |
| 013433198 | *Sarcophaga (Sarcophaga) crassipalpis* | Male | 2018 | South Kensington, London |
| 013433199 | *Sarcophaga (Sarcophaga) crassipalpis* | Male | 2018 | South Kensington, London |
| 010397298 | *Sarcophaga (Helicophagella) agnata* | Female | 1933 | Wyre Forest |
| 010397354 | *Sarcophaga (Heteronychia) pumila* | Female | 1951 | Hog's Back, Surrey |
| 010397359 | *Sarcophaga (Heteronychia) pumila* | Female | 1899 | St. Ives |
| 010579717 | *Sarcophaga (Helicophagella) melanura* | Female | 1953 | Oxwich |
| 010397370 | *Sarcophaga (Helicophagella) melanura* | Female | 1953 | Berrow |
| 010397414 | *Sarcophaga (Liosarcophaga) teretirostris* | Female | 1933 | Sussex |
| 010397415 | *Sarcophaga (Liosarcophaga) teretirostris* | Female | 1938 | Beachy Head |
| 010397432 | *Sarcophaga (Heteronychia) vagans* | Female | 2013 | Lancashire |
| 010397433 | *Sarcophaga (Heteronychia) vagans* | Female | 1912 | Wormsley |
| 010241250 | *Sarcophaga (Sarcophaga) variegata* | Female | 1931 | Aston Rowant |


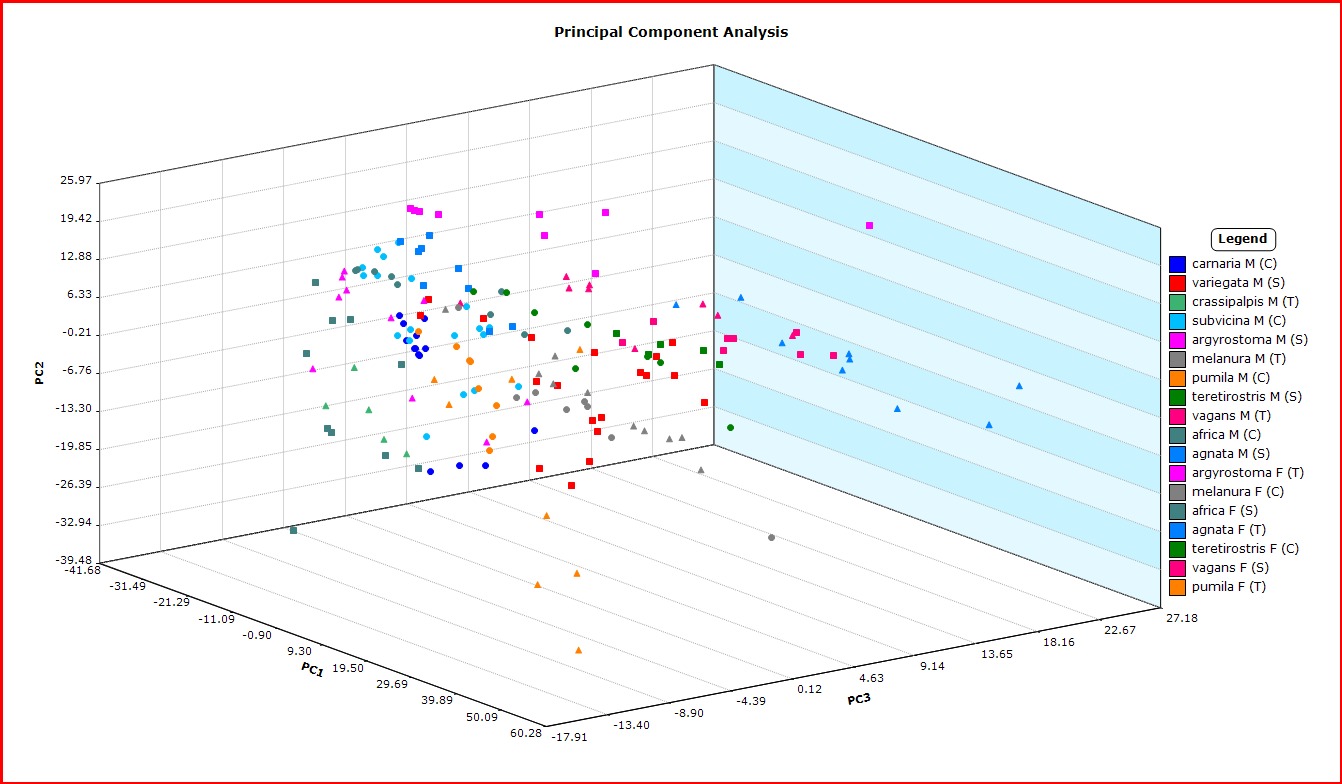


Figure S1: Principal component analysis calculated using the covariance matrix for samples from the all species and genders (M =Males, F = Females; C = Circles, S = Squares, T = Triangles). The first three principal components cover 67.2%, 13.3% and 6.2% of the variance respectively for a total of 86.7% variance. The three-dimensional PCA plot for all classes and genders is complex and does not permit easy visual separation of all classes.
